# Supplementary material for: A scoping review of barriers and facilitators to implementing tele-mental health solutions for rural, remote and underserved populations in low- and middle-income countries
Source: PLOS Digit Health. 2026 Jun 25;5(6):e0000903. doi: 10.1371/journal.pdig.0000903 (PMC13298784; doi:10.1371/journal.pdig.0000903)
Supplement: S1 Text — (DOCX) [file pdig.0000903.s002.docx]

## **S1 Text: Example of detailed search strategy**

Search: **Telemedicine AND low- and middle-income countries AND Mental health AND challenges** Filters: **in the last 10 years**

(("telemedicine"[MeSH Terms] OR "telemedicine"[All Fields] OR "telemedicine s"[All Fields]) AND ("developing countries"[MeSH Terms] OR ("developing"[All Fields] AND "countries"[All Fields]) OR "developing countries"[All Fields] OR ("low"[All Fields] AND "middle"[All Fields] AND "income"[All Fields] AND "countries"[All Fields]) OR "low and middle income countries"[All Fields]) AND ("mental health"[MeSH Terms] OR ("mental"[All Fields] AND "health"[All Fields]) OR "mental health"[All Fields]) AND ("challenge"[All Fields] OR "challenged"[All Fields] OR "challenges"[All Fields] OR "challenging"[All Fields])) AND (y_10[Filter])

Search: **Virtual care AND low- and middle-income countries AND Mental health AND challenges** Filters: **in the last 10 years**

(("virtual"[All Fields] OR "virtuality"[All Fields] OR "virtualization"[All Fields] OR "virtualized"[All Fields] OR "virtualizing"[All Fields] OR "virtuals"[All Fields]) AND "care"[All Fields] AND ("developing countries"[MeSH Terms] OR ("developing"[All Fields] AND "countries"[All Fields]) OR "developing countries"[All Fields] OR ("low"[All Fields] AND "middle"[All Fields] AND "income"[All Fields] AND "countries"[All Fields]) OR "low and middle income countries"[All Fields]) AND ("mental health"[MeSH Terms] OR ("mental"[All Fields] AND "health"[All Fields]) OR "mental health"[All Fields]) AND ("challenge"[All Fields] OR "challenged"[All Fields] OR "challenges"[All Fields] OR "challenging"[All Fields])) AND (y_10[Filter])

Search: **Virtual care AND low- and middle-income countries AND Mental health AND opportunities** Filters: **in the last 10 years**

(("virtual"[All Fields] OR "virtuality"[All Fields] OR "virtualization"[All Fields] OR "virtualized"[All Fields] OR "virtualizing"[All Fields] OR "virtuals"[All Fields]) AND "care"[All Fields] AND ("developing countries"[MeSH Terms] OR ("developing"[All Fields] AND "countries"[All Fields]) OR "developing countries"[All Fields] OR ("low"[All Fields] AND "middle"[All Fields] AND "income"[All Fields] AND "countries"[All Fields]) OR "low and middle income countries"[All Fields]) AND ("mental health"[MeSH Terms] OR ("mental"[All Fields] AND "health"[All Fields]) OR "mental health"[All Fields]) AND ("opportune"[All Fields] OR "opportunities"[All Fields] OR "opportunity"[All Fields])) AND (y_10[Filter])

Search: **Mhealth AND mental health AND low- and middle-income countries AND challenges AND opportunities** Filters: **in the last 10 years**

(("mhealth s"[All Fields] OR "telemedicine"[MeSH Terms] OR "telemedicine"[All Fields] OR "mhealth"[All Fields]) AND ("mental health"[MeSH Terms] OR ("mental"[All Fields] AND "health"[All Fields]) OR "mental health"[All Fields]) AND ("developing countries"[MeSH Terms] OR ("developing"[All Fields] AND "countries"[All Fields]) OR "developing countries"[All Fields] OR ("low"[All Fields] AND "middle"[All Fields] AND "income"[All Fields] AND "countries"[All Fields]) OR "low and middle income countries"[All Fields]) AND ("challenge"[All Fields] OR "challenged"[All Fields] OR "challenges"[All Fields] OR "challenging"[All Fields]) AND ("opportune"[All Fields] OR "opportunities"[All Fields] OR "opportunity"[All Fields])) AND (y_10[Filter])
