# Supplementary material for: A scoping review of barriers and facilitators to implementing tele-mental health solutions for rural, remote and underserved populations in low- and middle-income countries
Source: PLOS Digit Health. 2026 Jun 25;5(6):e0000903. doi: 10.1371/journal.pdig.0000903 (PMC13298784; doi:10.1371/journal.pdig.0000903)
Supplement: S1 Table — (DOCX) [file pdig.0000903.s003.docx]

## **S1 Table: World Health Organization regional classification of countries**

| **Region** | **Country** | **Number** | **First author** |
| --- | --- | --- | --- |
| Africa |  | 1 | Ibragimov et al |
|  | Ghana | 1 | Estapé et al., 2022 |
|  | Kenya | 1 | Estapé et al., 2022 |
|  | Nigeria | 1 | Estapé et al., 2022 |
|  | South Africa | 1 | Clough et al., 2017 |
|  | Uganda | 1 | Estapé et al., 2022 |
|  | Zambia | 1 | Munthali-Mulemba et al., 2022 |
| Americas | Argentina | 1 | Estapé et al., 2022 |
|  | Bolivia | 1 | Estapé et al., 2022 |
|  | Brazil | 1 | Estapé et al., 2022 |
|  | Chile | 1 | Estapé et al., 2022 |
|  | Colombia | 1 | Estapé et al., 2022 |
|  | Dominican Republic | 1 | Estapé et al., 2022 |
|  | Ecuador | 1 | Estapé et al., 2022 |
|  | Jamaica | 1 | Estapé et al., 2022 |
|  | Mexico | 1 | Estapé et al., 2022 |
|  | Peru | 1 | Estapé et al., 2022 |
| Eastern Mediterranean |  | 1 | Ibragimov et al |
|  | Iran | 1 | Clough et al., 2017 |
|  | Iraq | 1 | Knaevelsrud et al., 2014 |
|  | Jordan | 1 | Estapé et al., 2022 |
| Europe |  |  |  |
|  | Croatia | 1 | Estapé et al., 2022 |
|  | Lithuania | 1 | Estapé et al., 2022 |
|  | North Macedonia | 1 | Estapé et al., 2022 |
|  | Serbia | 1 | Estapé et al., 2022 |
| South-East Asia | Bangladesh | 1 | Koly et al., 2022 |
|  | India | 3 | Bhat et al., 2020  Estapé et al., 2022  Ganesh et al., 2022 |
|  | Nepal | 2 | Estapé et al., 2022  Bhardwaj et al., 2020 |
|  | Taiwan | 1 | Estapé et al., 2022 |
|  | The Philippines | 1 | Clough et al., 2017 |
|  | Turkey | 1 | Estapé et al., 2022 |
| Western Pacific | China | 1 | Estapé et al., 2022 |
