# Supplementary material for: A scoping review of barriers and facilitators to implementing tele-mental health solutions for rural, remote and underserved populations in low- and middle-income countries
Source: PLOS Digit Health. 2026 Jun 25;5(6):e0000903. doi: 10.1371/journal.pdig.0000903 (PMC13298784; doi:10.1371/journal.pdig.0000903)
Supplement: Attachment — Submitted filename: PDIG-D-25-00312_reviewer comments final.pdf [file pdig.0000903.s004.pdf]

PLOS Digital Health

A scoping review of barriers and facilitators to implementing tele-mental health solutions for rural, remote and underserved populations in low- and middle-income countries

--Manuscript Draft--

Manuscript Number:	PDIG-D-25-00312
Article Type:	Research Article
Full Title:	A scoping review of barriers and facilitators to implementing tele-mental health solutions for rural, remote and underserved populations in low- and middle-income countries
Short Title:	A scoping review of barriers and facilitators to implementing tele-mental health in LMICs
Corresponding Author:	Sandra Jumbe, CPsychol, fHEA<br>Queen Mary University of London Faculty of Medicine and Dentistry<br>London, London UNITED KINGDOM OF GREAT BRITAIN AND NORTHERN IRELAND
Order of Authors:	Sandra Jumbe, CPsychol, fHEA
	Aysha Groen
	Lisa M Ballard
	Alexander Torbuck
	Deniz Naghibi
	Sophie Leijdesdorff
	Jose G Perez-Ramos
	David Lim
	Virender Sorout
	Dingaane Msowoya
	Winnie W. S. Mak
	Rodrigo Ramalho
	Bessie Malila
Keywords:	mental health, tele-mental health, telepsychiatry, digital intervention, underserved communities, scoping review, low-middle-income countries
Abstract:	The increasing mental health gap in low- and middle-income countries (LMICs) poses a great global public health concern. Access to mental healthcare remains challenging due to stigma, lacking quality treatment options, and skilled workforce shortages. Tele-mental health involving remote access to care using information and communications technologies supported by mobile communication systems may be a promising solution. However, most research in this area has been conducted in high-income countries. This scoping review aimed to explore known barriers and facilitators to implementing tele-mental health solutions to populations in remote, rural, and underserved communities in LMICs to inform evidence-based development and implementation of digital interventions and strategies that can improve access to quality and affordable mental healthcare.<br>Studies published between 2010 and 2024 were identified through comprehensive searches across four databases, namely PubMed, PsycINFO, Web of Science, and SpringerLink. Nine studies were included for analysis. Barriers and facilitators were categorised using the Capability, Opportunity, and Motivation = Behaviour (COM-B) model. A narrative synthesis revealed several barriers for patients, primarily related to limited physical and social opportunities, such as lack of mobile devices, unstable or limited internet access, and lack of private space at home. Barriers for mental health

	providers were predominantly linked to psychological capability, including insufficient knowledge concerning tele-mental health interventions. Time and cost efficiencies were key facilitators for both service users and mental health service providers, which enhanced access to care. Infrastructure development, educational initiatives, and training for providers are essential to create a more equitable tele-mental healthcare system.
**Additional Information:**	
**Question**	**Response**
**Government Employee**<br><br>Are you or any of the contributing authors an employee of the United States government?<br><br>Manuscripts authored by one or more US Government employees are not copyrighted, but are licensed under a CC0 Public Domain Dedication, which allows unlimited distribution and reuse of the article for any lawful purpose. This is a legal requirement for US Government employees.<br><br>This will be typeset if the manuscript is accepted for publication.	No - No authors are employees of the U.S. government.
**Financial Disclosure**<br><br>Enter a financial disclosure statement that describes the sources of funding for the work included in this submission. Review the submission guidelines for detailed requirements. View published research articles from *PLOS Digital Health* for specific examples.<br><br>This statement is required for submission and **will appear in the published article** if the submission is accepted. Please make sure it is accurate.	Yes

**Funded studies**<br>Enter a statement with the following details:<br>• Initials of the authors who received each award<br>• Grant numbers awarded to each author<br>• The full name of each funder<br>• URL of each funder website<br>• Did the sponsors or funders play any role in the study design, data collection and analysis, decision to publish, or preparation of the manuscript?<br><br>Did you receive funding for this work?	
Please add funding details.<br>as follow-up to "**Financial Disclosure**<br><br>Enter a financial disclosure statement that describes the sources of funding for the work included in this submission. Review the submission guidelines for detailed requirements. View published research articles from *PLOS Digital Health* for specific examples.<br><br>This statement is required for submission and **will appear in the published article** if the submission is accepted. Please make sure it is accurate.<br><br>**Funded studies**<br>Enter a statement with the following details:<br>• Initials of the authors who received each award<br>• Grant numbers awarded to each author<br>• The full name of each funder<br>• URL of each funder website<br>• Did the sponsors or funders play any role in the study design, data collection and analysis, decision to publish, or preparation of the manuscript?<br><br>Did you receive funding for this work?"	This work is supported by the Worldwide Universities Network – Research Development Fund 2023 awarded to Dr Bessie Malila. Lisa Ballard's time was funded/supported by the National Institute for Health and Care Research (NIHR) Southampton Biomedical Research Centre. Bessie Malila and Sandra Jumbe's time was also funded through the African Research Initiative for Scientific Excellence (ARISE) pilot programme (Grant No. DCI-PANAF/2020/420-028) implemented by the African Academy of Sciences with support from the European Commission and the African Union Commission. The views expressed are those of the author(s) and not those of the listed funders.
Please select the country of your main research funder (please select carefully as in some cases this is used in fee calculation).	UNITED KINGDOM - GB

as follow-up to "**Financial Disclosure**<br><br>Enter a financial disclosure statement that describes the sources of funding for the work included in this submission. Review the submission guidelines for detailed requirements. View published research articles from *PLOS Digital Health* for specific examples.<br><br>This statement is required for submission and **will appear in the published article** if the submission is accepted. Please make sure it is accurate.<br><br>**Funded studies**<br>Enter a statement with the following details:<br>• Initials of the authors who received each award<br>• Grant numbers awarded to each author<br>• The full name of each funder<br>• URL of each funder website<br>• Did the sponsors or funders play any role in the study design, data collection and analysis, decision to publish, or preparation of the manuscript?<br><br>Did you receive funding for this work?"	
**Competing Interests**<br><br>On behalf of all authors, disclose any competing interests that could be perceived to bias this work.<br><br>This statement will be typeset if the manuscript is accepted for publication.<br><br>Review the instructions link below and PLOS Digital Health's competing interests policy to determine what information must be disclosed at submission.	All authors declare no competing interests
**Data Availability**<br><br>Provide a **Data Availability Statement** in	All data are in the manuscript and supporting information files.

the box below. This statement should detail where the data used in this submission can be accessed. This statement will be typeset if the manuscript is accepted for publication.<br><br>Before publication, authors are required to make all data underlying their findings fully available, without restriction. Review our PLOS Data Policy page for detailed information on this policy. Instructions for writing your Data Availability statement can be accessed via the Instructions link below.	

Powered by Editorial Manager® and ProduXion Manager® from Aries Systems Corporation

Manuscript

Click here to access/download;Manuscript;WUN scoping review manuscript 20.5.25.docx

A scoping review of barriers and facilitators to implementing tele-mental health solutions for rural, remote and underserved populations in low- and middle-income countries

Sandra Jumbe[1,9], Aysha Groen[2], Lisa Ballard[5], Alexander Torbuck[3], Deniz Naghibi[3], Sophie Leijdesdorff[2a], Jose G Perez-Ramos[3], David Lim[6], Virender Sorout[6], Dingaane Msowoya[1], Winnie WS Mak[7], Rodrigo Ramalho[8], Bessie Malila[4]

1. School of Social and Health Sciences, Millennium University, Blantyre, Malawi.
2. Department of Psychiatry and Neuropsychology, The Mental Health and Neuroscience Research Institute[2a], Maastricht University, Maastricht, the Netherlands.
3. Department of Public Health Sciences, University of Rochester, School of Medicine and Dentistry, Rochester, New York, USA.
4. Division of Biomedical Engineering, Faculty of Health Sciences, University of Cape Town, Cape Town, South Africa.
5. National Institute for Health and Care Research (NIHR) Southampton Biomedical Research Centre. School of Primary Care, Population Sciences and Medical Education, Primary Care Research Centre, University of Southampton, Southampton, United Kingdom.
6. Faculty of Health, University of Technology Sydney (UTS), Broadway, Sydney, Australia.
7. Department of Psychology, The Chinese University of Hong Kong, Shatin, NT, Hong Kong.
8. Department of Social and Community Health, School of Population Health, University of Auckland, Auckland, New Zealand.
9. Centre for Evaluation and Methods, Wolfson Institute of Population Health, Queen Mary University of London, London, United Kingdom

Word count = 5538 (abstract and manuscript excluding tables, figures and references)

Abstract

The increasing mental health gap in low- and middle-income countries (LMICs) poses a great global public health concern. Access to mental healthcare remains challenging due to stigma, lacking quality treatment options, and skilled workforce shortages. Tele-mental health involving remote access to care using information and communications technologies supported by mobile communication systems may be a promising solution. However, most research in this area has been conducted in high-income countries. This scoping review aimed to explore known barriers and facilitators to implementing tele-mental health solutions to populations in remote, rural, and underserved communities in LMICs to inform evidence-based development and implementation of digital interventions and strategies that can improve access to quality and affordable mental healthcare.

Studies published between 2010 and 2024 were identified through comprehensive searches across four databases, namely PubMed, PsycINFO, Web of Science, and SpringerLink. Nine studies were included for analysis. Barriers and facilitators were categorised using the Capability, Opportunity, and Motivation = Behaviour (COM-B) model. A narrative synthesis revealed several barriers for patients, primarily related to limited physical and social opportunities, such as lack of mobile devices, unstable or limited internet access, and lack of private space at home. Barriers for mental health providers were predominantly linked to psychological capability, including insufficient knowledge concerning tele-mental health interventions. Time and cost efficiencies were key facilitators for both service users and mental health service providers, which enhanced access to care. Infrastructure development, educational initiatives, and training for providers are essential to create a more equitable tele-mental healthcare system.

Keywords: mental health, tele-mental health, telepsychiatry, digital intervention, underserved communities, scoping review, low-middle-income countries

Background

The escalating prevalence of poor mental health, particularly in low- and middle-income countries (LMICs), poses a significant concern (1). Eighty two percent of the global population struggling with at least one diagnosable health condition live in LMICs (2, 3). Common mental health challenges such as depression and anxiety are leading causes of disease burden (4-6). Social Determinants of Health such as low income, low education, and social exclusion are key contributors to mental health challenges (4, 7). The prevalence of common mental illnesses like depression and anxiety, have been exacerbated by the COVID-19 pandemic, the effects of climate change, conflicts and wars (1, 8). These factors have had both a direct negative impact on mental health due to stress and trauma, as well as indirect negative impacts on various socio-economic aspects, contributing to the vicious cycle of poverty in LMICs (3, 4).

Access to appropriate mental health care remains a challenge in LMICs for various reasons. Firstly, there is often stigma and misunderstanding surrounding mental health, causing individuals to not seek help and avoid available services (2, 9). Secondly, mental health is often not seen as a priority within the healthcare system (9), which results in limited government funding, lack of evidence-based treatment and care options (4, 10), and persistent acute shortages of skilled mental health professionals and limited resources and support for these professionals (11, 12). In many LMICs, less than 1% of the total health budget is allocated to mental health leaving many areas without adequate mental health services; with geographically remote and rural areas being more disproportionately neglected (1, 9). As a result, approximately 85% of individuals in LMICs with mental health problems remain untreated (13-15). Untreated mental illness can precipitate other chronic health conditions, leading to reduced social cohesion and elevated risk of behavioural problems, which further feed into social inequities (16, 17). These aspects have negative economic repercussions

leading to higher healthcare costs, diminished labour productivity, and increased utilisation of health and social services (18, 19).

Tele-mental health (also known as tele-psychiatry) refers to remote provision of mental healthcare, utilising technologies such as telephone calls, video conferencing, online platforms, the Internet of Things, mobile and other applications, including generative Artificial Intelligence (21). Telehealth was originally developed to provide basic care to rural and underserved patients (20). The importance of telehealth has been amplified since the COVID-19 pandemic, becoming standard practice in many settings and influencing recent legislative initiatives to advocate for more telehealth advancements to improve health service provision (20). Tele-mental health can reduce the cost of care by eliminating travel expenses for both specialist mental health care service providers and service users, especially those residing in remote or rural areas (21-23). It also reduces the need for physical infrastructure, such as clinical space and clinics, resulting in reduced overhead costs (24). Additionally, it has been shown to increase accessibility to evidence-based care from specialised healthcare providers, offering flexibility in treatment options and facilitating more efficient use of time and resources for both healthcare providers and users (22, 25). These advantages can lead to positive spillover effects on physical health and help prevent costly Emergency Department utilisation, hospitalisations, avoidable readmissions, and long-term treatment expenses (26, 27).

Although tele-mental health shows promise to enhance access to quality affordable mental healthcare, it also presents unique challenges. Privacy concerns, technological barriers, and the need for appropriate training and quality assurance for healthcare providers are among these challenges (8). Additionally, the remote nature of tele-mental health may overlook certain behavioural cues, while limited internet access may contribute to digital exclusion, hence further hindering its accessibility and effectiveness (8, 28). Nevertheless, it remains a growing and evolving field that can be a valuable complement to traditional face-to-face mental healthcare (29).

Most tele-mental health studies to date have been conducted in high-income countries (30). Research also unveiled a significant imbalance in the distribution of mental health resources globally. Despite 82% of diagnosable mental health conditions occurring in LMICs, more than 90% of the available mental health resources are concentrated in high-income countries (11, 31). This stark contrast highlights a critical disparity in the mental health treatment gap between LMICs and high-income countries. While LMICs bear the brunt of the mental health burden, they are disproportionately underserved in terms of resources to address these challenges; Individuals living in remote and rural areas, and those who are disadvantaged, marginalised and vulnerable are particularly deprived (32-34). This imbalance highlights the urgent need for solutions that promote fair and equitable access to mental health services in LMICs.

To date, there is no comprehensive review of digital solutions that can deliver mental healthcare to rural, remote and underserved communities in LMICs. In this context, examples of underserved communities include caregivers, disabled, ethnic minorities, LGBT+, youth, and older populations. There is also no review focusing on specific barriers and facilitators to delivering tele-mental health interventions (TMHIs), specifically among rural, remote and underserved communities in LMICs. This review summarised existing evidence on TMHIs for rural, remote and underserved communities in LMICs, with a particular focus on identifying reported barriers and facilitators to their implementation. The expected outcome of this work is to provide comprehensive evidence to inform the development and implementation of tele-mental health solutions for remote, rural, and underserved communities.

Method

Search strategy

This scoping review was conducted in accordance with the Preferred Reporting Items for Systematic Reviews and Meta-Analyses extension for scoping reviews (PRISMA-ScR) (35). The PRISMA-ScR checklist can be found in Appendix 1.

Published empirical studies were included that focused on TMHIs conducted in LMICs among individuals living in remote, rural and underserved communities. LMICs were classified according to the World Bank definition (36). Qualitative and quantitative studies with different study designs were included to provide a comprehensive understanding of the topic. Publications in English, Dutch, Cantonese Chinese, Spanish, and French were considered as our review team comprised individuals from diverse backgrounds who collectively spoke these languages. Table 1 details the inclusion and exclusion criteria.

Table 1: Scoping review eligibility criteria

Inclusion criteria	Exclusion criteria
▪ Empirical studies on tele-mental health interventions or applications utilising technologies such as telephone calls, video conferencing, online platforms, and mobile applications.<br>▪ Studies conducted in LMICs according to the World Bank definition.<br>▪ Intervention delivered in remote, rural, and/ or underserved communities.<br>▪ Qualitative and/or quantitative study design. This includes randomised controlled trials, non-randomised controlled trials, time-series studies, analytical observational studies, cohort studies, case-control studies, cross-sectional studies and qualitative studies<br>▪ Studies published since January 2010.	▪ Non empirical evidence, e.g., commentaries, opinion pieces, and unpublished grey literature.<br>▪ Literature, scoping or systematic reviews; however, we hand-searched reference lists of reviews for relevant studies.<br>▪ Papers studying mental health unrelated to tele-mental health solutions or interventions.<br>▪ Studies not explicitly focused on delivering tele-mental health solutions or interventions to remote, rural and underserved communities (as reported by authors).<br>▪ Studies conducted outside of a LMIC.<br>▪ Studies published before January 2010.

Three reviewers (SJ, AG and LB) developed the search strategy (Table 2 and Appendix 2) guided by the PICO framework for scoping reviews which concentrates on population, concept, and context (37). We included studies published since January 2010 as this timeframe

captured literature on recent advancements and trends in tele-mental health, reflecting current technology and practice. The last search was conducted on June 18, 2024.

Table 2: Search strategies used within the scoping review

PICO elements	Definition	Search string (synonyms connected by OR)
Population (key participant characteristics)	Remote, rural and/or underserved communities	Remote, rural, underserved community, communities
Concept (intervention and outcomes)	Mental health intervention	Therapy, intervention, treatment, mental healthcare, psychological support, counselling, psychotherapy
	Barrier	Barrier, challenge, hindrance, constraint, difficulty, limitation
	Facilitator	facilitator, enabler, access, utilisation, opportunity, adherence
Context	Digitally delivered	Digital, m-Health, online app, mobile app, internet, e-health, computer, web-based, tele-mental health, telehealth, telemedicine, virtual care, virtual clinic
	Living in low and middle-income countries	LMIC, low and middle-income countries, developing countries, resource-limited, resource-poor

Screening and selection of evidence

Articles were selected in three phases: (i) identification, (ii) screening, and (iii) inclusion, according to PRISMA guidelines. Primary searches of PubMed, PsycINFO, Web of Science, and SpringerLink databases identified 754 articles. The literature identified by the specified search terms was imported to EndNote (version 20). After manual title screening of these 754 articles by two reviewers (AG and DM), 537 studies were excluded mainly because their title did not include a tele-mental health intervention (Figure 1).

The remaining list of 217 studies was imported into Rayyan, a web-based systematic review management tool (38) for abstract screening according to the inclusion and exclusion criteria by AG and DM under the guidance of SJ. At this point, 15 duplicate citations were removed, and 147 studies were excluded, leaving 55 studies for full paper screening. The interrater reliability between the reviewers assessed by Rayyan was 81%. Discrepancies between the two independent reviewers were reviewed independently by a third reviewer (SJ) and resolved collaboratively.

Full-text screening of the 55 papers was carried out by four reviewers (AG, AT, DN and VS), split into pairs, using the data extraction form for each paper (Table 3). Disagreements between reviewers were resolved collaboratively during weekly meetings led by SJ. 47 papers were excluded, leaving eight included studies. An additional study was identified through a hand search of the 55 papers' reference lists, resulting in a total of nine included papers in this review. Reasons for exclusion for each article were recorded accordingly (see Figure 1). Key characteristics of these nine included studies are listed in Table 3.

Table 3: Characteristics of the Included Studies

First Author	Study Design	Setting	Disparity Groups	Sample size	Age	Gender	Country/ Regions	Mental Health Condition	Intervention	Barriers	Facilitators
Ibragimov et al., 2022	Mixed methods	Rural	Migrants, Urban population, Long-term chronic disease, Maternal Health Program, Surgical/ burns patients, Paediatric patients	81	n/a	n/a	44 countries located in Africa, The Middle East, Asia	Any mental health condition regardless of severity	Audio-only platforms (Phone calls & Voice chat software), Video conferencing	Internet access, Internet and cell phone connections, Access to communication devices, Private space, Thought staff had a negative or mixed impression, Increased workload, Staff knowledge, Cultural barriers, Non-verbal cues	Improved access, Cost-effective for patients and staff, Time effective for patients and staff, Safety strategies, Increased engagement, Staff adaptability, Positive attitude against tele-mental health
Clough et al., 2017	Cross-sectional survey	Urban	n/a	524	Mean age: 29.9 Range: 18-80	25.6% Male, 74.40% Female	Iran, the Philippines, South Africa	Difficulties with emotions, Use of alcohol or drugs	Video conferencing, Text messaging (Chat & E-mail), Self-help module, Information websites	Internet access, Access to communication devices, Private space, Personal information, Lack of information	willingness to use Tele-mental health
Bhat et al., 2020	Qualitative study	Rural	Rural Indian women	69	Mean age: 46.65 Range: 22-81	100% Female	India	Major Depressive Disorder	Audio-only platforms (Phone calls), Text messaging (Chat)	Mobile network, Access to communication devices, Private space, Community stigma, Illiteracy	Improved access, Cost-effective, Time effective
Estapé et al., 2022	Cross-sectional survey study	Both	Caregivers	91	Mean: 42,9 Range: 21-70	21.1% Male, 78.8% Female	25 countries located in Africa, South and Central America, Eastern	Distress, Anxiety, Depression	Audio-only platforms (Phone calls), Video conferencing,	Access to communication devices, Staff knowledge	Staff adaptability, Interested in receiving training, Felt qualified to

							Mediterranean, Europe, South-East Asia, Western Pacific		Text messaging (Chat)		deliver tele-mental health, Positive attitude against tele-mental health
Munthali-Mulemba et al.,2022	Qualitative study	Urban	Adolescents, Young adults	17	Mean age: n/a Range: 15-29	50% Male, 50% Female	Zambia	Traumatic experiences, Substance use, Physical/ sexual violence, Anxiety	Audio-only platforms (Phone calls)	Internet connections, Access to communication devices, Electricity, Interrupting calls, Private space	Improved access, cost-effective, time effective, reduced other responsibility, Safety strategies, feelings of engagement and comfort, Information to overcome barriers, better communication with teams, punctuality and consistency
Koly et al., 2022	Qualitative	Rural	n/a	37	n/a	27% Male, 73% Female	Bangladesh	Anxiety, panic attack, suicidal thoughts, Depression, Substance abuse disorders	Video conferencing, Information websites, Digital photos, Social media	Internet access, Internet connections, Access to communication devices, Private space, Personal information, Community stigma, Staff knowledge, Non-verbal cues, Prescribing psychiatric medications	Improved access, cost-effective for patients and staff, time effective, Information to overcome barriers, staff positive attitude against tele-mental health
Ganesh et al., 2022	Case study	Both	n/a	2401	n/a	n/a	India	n/a	Audio-only platforms (Phone calls),	Internet connections, Mobile network,	Improved access, cost-effective, time

									Video conferencing, Text messaging (Chat & SMS)	Non-verbal cues, Prescribing psychiatric medications	effective for patients and staff
Bhardwaj et al., 2020	Mixed-methods study	Rural	Community health workers	36	Mean age: 46,9 Range:30	100% Female	Nepal	Depression, Postpartum depression, Alcohol use disorder, Psychosis	Text messaging (SMS)	Internet connections, Personal information, Community stigma, Staff knowledge, Staff confidence	Positive attitude against tele-mental health, better communication with teams
Knaevelsrud et al., 2014	Randomized Controlled Trial	Rural	Adults with PTSD	159	Mean age: 33.5 Range: 18-56	28.3% Male, 71.7% Female	Iraq, other Arab nations	Post-traumatic stress disorder	Video conferencing, Text messaging (Chat), Self-help module	Internet access, Internet connections, Electricity, Private space, Cultural barriers	Improved access, staff adaptability

Note: N = Total number of participants.

Data extraction and analysis

Key information from the included studies such as study characteristics, key findings, and relevant outcomes was recorded on the data extraction form developed collectively by the review team (Table 3). This data included specific details about the participants, intervention (e.g. tele-mental health solution), context (e.g. country), study methods and key findings. We piloted using the data extraction form tool to ensure that it would capture key aspects needed to answer our scoping review questions. Specifically, we randomly selected five papers from the preliminary search results and used the tool to independently extract data. The team subsequently revised and agreed on specific fields to include in the data extraction tool.

A narrative synthesis was conducted to outline the spectrum of tele-mental health solutions described across included studies. Narrative synthesis involves summarising and interpreting the findings from multiple studies to provide a coherent and comprehensive overview of the evidence, often identifying patterns, themes, and variations within the data (39). This approach was supported by a detailed tabular presentation using the data extraction tool. Through this inductive analysis, common trends and regional variations in the utilisation of tele-mental health solutions, unique themes and characteristics of participant demographics were identified.

The data on barriers and facilitators affecting tele-mental health implementation was subsequently deductively coded to the Capability, Opportunity, and Motivation = Behaviour (COM-B) model (40), which categorises influencing factors into six domains within its theoretical framework. In the COM-B model for behaviour change, capability (C), opportunity (O), and motivation (M) are cited as three key factors capable of changing behaviour (B) (40, 41). Capability refers to an individual’s physical and psychological capacity to engage in an activity. Opportunity encompasses external factors that facilitate or hinder behaviour, such as environmental and social influences. Motivation includes both reflective and automatic mechanisms that activate or inhibit the behaviour.

The frequency of identified barriers and facilitators was reported, and narrative synthesis was employed within each domain to deepen our understanding of specific factors influencing access to TMHIs as reported by authors of the included studies. Finally, the COM-B model was applied to derive key policy and behavioural intervention recommendations based on the findings (40).

Results

Characteristics of included studies

As shown in Table 3, the studies included in this scoping review were three qualitative studies (42-44), two mixed-methods studies (22, 45), two cross-sectional survey studies (46, 47), one randomised controlled trial (48), and one case study (49). All were written in English and were published between 2014 and 2022. Both service users and/or tele-mental health care providers' perspectives on barriers and facilitators were represented in seven studies (22, 43-45, 47-49). The remaining two studies, namely Bhat et al., 2020 (42) and Clough et al., 2017 (46), focused solely on barriers and facilitators of service users. Five studies focused on a single country (42-45, 49). The other four studies were multi-country studies (22, 46-48). These multi-country studies had cohorts from across the globe. For example, the study by Clough et al (2017) had participants from South-East Asia, Africa and the Eastern Mediterranean (46). One study included participants from The Americas, Europe and Western Pacific Region (47). The 30 identified countries amongst the included studies are categorised into regions using the World Health Organisation's classifications in Appendix 3.

Four studies took place in rural communities (22, 42, 43, 45). Rural areas were described as villages in two studies (42, 45) whilst the other two provided no specific definitions for rurality (22, 43). Three studies were conducted in urban areas (44, 46, 48), and two in both rural and urban settings (47, 49). Additionally, six studies focused on an underserved community (22, 42, 44, 45, 47, 48).

The average number of participants across the included studies was 379, with sample sizes ranging from 17 to 2401. Age of participants was reported in six studies (42, 44-48); the mean participant age being 36 years, with ages ranging from 15 to 81 years. Two studies, namely Bhardwaj et al., 2020 and Bhat et al., 2020, included only female participants (42, 45). Although the other five studies included both male and female participants, most of these studies had more females. Two studies did not disclose participant gender (22, 49).

Among the included studies, seven described multiple TMHIs, while Bhardwaj et al. (2020) and Munthali-Mulemba et al (2022) focused on one . Text messaging (chat, SMS and e-mail) was the most common intervention, used in seven studies (22, 42, 45-49), with chat used in six studies (22, 42, 46-49), followed by SMS in two studies (45, 49), and e-mail in one study (46). Videoconferencing was also used in six studies (22, 43, 46, 47). Audio-only platforms (phone calls or voice chat software) were mentioned in five studies (22, 42, 44, 47, 49). Of these audio-only platforms, all five studies used telephone calls and only one study by Ibragimov et al. (2022) used voice chat software (22). Two studies used informational websites (43, 46) and self-help modules with or without online therapist assistance (46, 48). Digital photos and social media featured in one study (43).

Various TMHIs were implemented for different mental health conditions. The most commonly mentioned conditions were major depressive disorder (42, 43, 45-47) and substance use disorders (43-46). Three studies reported anxiety (43, 44, 47). Two studies mentioned traumatic experiences (44, 48), such as physical or sexual violence, with Knaevelsrud et al., (2014) specifically addressing post-traumatic stress disorder as a consequence. Suicidal thoughts were mentioned in two studies (43, 45) and other conditions mentioned in one study included difficulties with emotions, distress, panic attacks, postpartum depression, and psychosis (45). Additionally, one study focused on every mental health condition, regardless of severity (22), while another study did not specify any mental health condition (49).

Key findings from included studies

Service user barriers

Table 4 summarises results from the COM-B analysis on barriers to tele-mental health solutions identified across the included studies. All nine studies mentioned barriers to TMHIs. Specifically, all nine studies included barriers concerning opportunities for service users. In terms of physical opportunities, it was frequently noted that patients often lacked their own communication devices or had to share or borrow devices from others to use TMHIs (22, 42-44, 46, 47). Consequently, they could not receive appointment reminders via text messages or phone calls. Sharing or borrowing communication devices also resulted in shortened or interrupted appointments. Unstable internet connections were also a common issue noted in six studies (22, 43-45, 48, 49), with four of these studies stating that patients had no internet access (22, 43, 46, 48) or experienced problems with their mobile phone network coverage (22, 42, 48, 49). Mobile phone signal was often location and time dependent; peak hours could lead to dropped calls, background noise, and other connection difficulties (22, 48).

Limited access to electricity to keep mobile phones charged was also mentioned in two studies as a barrier (44, 48), as well as the interruption of sessions by incoming calls (44). Social opportunities were another frequently cited barrier for users, with six out of the nine studies reporting that patients lacked private space at home (22, 42-44, 46, 48). Patients were afraid of being overheard or caught in mental health consultation assumedly due to stigma linked with mental illness. Some users shared their space or had children at home, interrupting their privacy during TMHIs consultations (42-44, 48). Additionally, family members or partners sometimes felt threatened by the relationship between the service user and the mental healthcare professional or feared that the service user might disclose certain sensitive information (22, 44, 48). This led to family members denying access to communication devices, joining sessions to monitor them or even being violent towards the service user.

Capability barriers were identified in two studies (42, 46). Psychological capability included patients lacking information about tele-mental health services (46). Moreover, one study found that those in remote areas were sometimes unable to read, which made understanding text messages challenging (42). In terms of reflective motivation, one study mentioned that patients believed their providers held negative or ambivalent attitudes towards tele-mental health (22). Service users also expressed concerns about the privacy of their personal information (43, 45, 46). Due to the stigma associated with mental health, individuals required assurances that their data was secure and treated confidentially (42, 43, 45). This need for trust was heightened by the fact that unsecured network connections lead to concerns about compromised confidentiality, thereby fostering mistrust in services. No barriers related to automatic motivation were identified in any study.

Mental healthcare provider barriers

A key barrier affecting the provision of tele-mental health services by mental healthcare providers in remote, rural, and/or underserved in LMICs was related to psychological capability (22, 43, 45, 47, 49). The lack of non-verbal cues during audio-only interventions adversely affected communication (22, 43, 49), resulting in communication gaps. Mental health care providers also reported difficulties in assessing patients' conditions due to the absence of non-verbal cues (49).

In terms of physical opportunity barriers, a notable issue around lacking knowledge regarding TMHIs was identified in four studies (22, 43, 45, 47), as providers faced challenges in transitioning from traditional to TMHIs formats because of the absence of clear protocols and guidelines. Additionally, two studies reported cultural barriers (22, 48), particularly language issues. Two studies mentioned the inability to prescribe certain psychiatric medications online for the treatment of severe mental health disorders due to prescription validity (43, 49). In Koly et al (2022), participants stated that this issue was due to restricted legislative ability as there were '*guidelines and* a' specific '*list of permitted drugs for online sessions from the Bangladesh*

College of Physicians and Surgeons', list of permitted drugs for online sessions from Bangladesh College of Physicians and Surgeons , which meant one could not prescribe medication outside of this list. Ibragimov et al. (2022) also mentioned barriers related to social opportunity, where tele-mental healthcare providers experienced an increased workload due to increased caseloads, 24/7 availability, and longer, more frequent sessions (22). Regarding motivation, one study highlighted reflective motivational issues, such as a lack of confidence in using technology (45). Barriers related to automatic motivation were not identified in the included studies.

Table 4: COM-B analysis of barriers to tele-mental health interventions

Component	Subcomponent	Patients	Mental healthcare Providers
Capability	Psychological capability	Lack of information about tele-mental health services	Lack of knowledge concerning eHealth interventions<br><br>Lack of non-verbal cues during audio-only interventions
Opportunity	Physical opportunity	Lack of access to the internet<br>Lack of or limited access to communication devices<br><br>Unstable internet connections<br>Poor mobile network coverage<br>Lack of or inconsistent electricity to keep devices charged<br>Interrupting incoming calls	Cultural barriers<br><br>Unable to prescribe all types of psychiatric medications online
	Social opportunity	Community stigma towards mental health<br>Concerns about personal information<br>Lack of a private space at home	Increased workload
Motivation	Reflective motivation	Believed that providers held a negative or ambivalent attitude toward tele-mental health	Lack of confidence using technology

Service user facilitators

Table 5 summarises the results of the COM-B analysis on facilitators of TMHIs. All nine studies identified facilitators of TMHIs. Opportunity was the most common facilitator for patients and was mentioned in six studies (22, 42-44, 48, 49). These studies also identified physical opportunities provided by TMHIs, such as improved access to care (22, 42-44, 48, 49). Time efficiency played a significant role in these five studies, with reductions in indirect costs such as travel time, time lost visiting clinics, and long distances walked (22, 42-44, 49). Cost-effectiveness was also noted in the same five studies, with reductions in travel-related costs. The use of TMHIs reduced other responsibility burdens, such as finding childcare (44). These factors provided service users with the necessary resources and settings to participate in tele-mental health services. Two studies identified facilitators related to social opportunities and highlighted implementation strategies to ensure service user's safety (22, 44). These strategies included scheduling sessions at times when the partner or family member is not home and agreeing on code words to use if someone enters the room.

Regarding capability, only psychological capability facilitators were identified (22, 43, 44). Two studies highlighted that obtaining information helped service users overcome barriers (43, 44). Providing information about tele-mental health-specific factors, such as privacy, confidentiality, finding a safe space, and maintaining a reliable connection, assisted users in understanding and building confidence in the privacy of the conversations and the confidentiality of their health information and data (44). Addressing community stigma towards mental health helped service users feel comfortable seeking mental healthcare remotely (43), creating a supportive environment that encouraged the use of tele-mental health services. Tele-mental health services also helped disseminate information, facilitating mass awareness of mental health symptoms and the various care options, and promoting self-referral when needed (43). One study noted increased engagement with tele-mental healthcare compared to traditional mental healthcare (22).

In terms of motivation, two studies discussed reflective motivation (44, 46). Munthali-Mulemba et al. (2022) reported that service users experienced feelings of engagement and comfort with their mental healthcare provider, even when communicating through audio-only platforms (44). Clough et al. (2017) noted that service users felt a strong willingness to use various tele-mental healthcare options, including audio-only platforms, video conferencing, text messaging, self-help modules, or information websites, preferably with assistance from an online therapist (46).

Mental healthcare provider facilitators

Facilitators for mental healthcare providers were predominantly around motivation. Six studies exclusively mentioned reflective motivation (22, 43-45, 47, 48). Specifically, mental healthcare providers recognised the positive impact of these interventions on their service users' lives, which led to reported positive attitudes towards tele-mental health (22, 43, 45, 47). This also reflected in their confidence and readiness to deliver this mode of service (47). Additionally, one study highlighted that mental healthcare providers wanted to receive training to enhance their ability to provide and participate in the development of TMHIs (47).

Three studies identified aspects linked to physical opportunity, reporting that TMHIs were time-management effective as they reduced travel-related time and allowed for more consultations (22, 49). Additionally, these interventions reduced travel-related costs (22, 43). Four studies identified facilitators in the context of psychological capability (22, 44, 47, 48). Three of these four studies noted that the adaptability of mental healthcare providers to transition to virtual care and their ability to offer a varied selection of TMHIs indicated that healthcare providers possess the necessary skills and knowledge to implement TMHIs effectively (22, 47, 48). One study stated that the punctuality and consistency of mental healthcare providers fostered feelings of comfort, trust, and engagement in TMHIs, thereby enhancing the development of a strong therapeutic alliance (44).

Table 5: COM-B Analysis of the Facilitators of Tele-mental Health Interventions

Component	Subcomponent	Service users	Mental healthcare providers
Capability	Physical capability	n/a	n/a
	Psychological capability	Information provided to overcome barriers<br>Increased engagement with tele-MH care	Staff adaptability to transition to virtual care<br>Punctuality and consistency of the providers enhance the establishment of a strong therapeutic alliance
Opportunity	Physical opportunity	Cost-effective<br><br>Time efficiency<br>Improved access to care<br>Reduced other responsibility burden	Cost-effective<br><br>Time efficiency
Motivation	Reflective motivation	Feelings of engagement and comfort with their provider	Positive attitude against tele-mental health
	Reflective motivation	Willingness to utilize tele-mental health services if provided	Recognition of the benefits for patients<br><br>Felt qualified to deliver tele-mental health intervention<br>Interested in receiving training

Discussion

Given the high prevalence of mental health conditions in LMICs, combined with significant treatment gaps, tele-mental health emerges as a promising alternative (1, 2). However, to date, no previous study has synthesised the evidence regarding the barriers and facilitators for the use of tele-mental health in LMICs, particularly regarding remote, rural, and underserved communities.

Main Findings

Extensive searches of four major databases yielded only nine studies that met the review's inclusion criteria, suggesting that research on tele-mental health solutions for rural, remote, and underserved communities in LMICs is still limited. This small number of studies may also

reflect broader issues of inadequate infrastructure and limited resources for research in LMICs to develop, implement, and publish evidence around digital health interventions. This gap in the literature highlights the need for further support to develop initiatives and conduct and disseminate research on tele-mental health in these regions.

Most studies were conducted in South-East Asia, Africa, and the Eastern Mediterranean region, suggesting geographical nonuniformity and highlighting a gap in research from LMICs in the Americas. Demographic insights of included studies revealed a predominantly female participant pool, which is consistent with existing literature suggesting that women are more likely to seek or receive mental health support (50, 51). The variety of TMHIs identified illustrated the versatility of this approach, with text messaging, audio-only platforms and videoconferencing emerging as the most prevalent modalities. Common mental health disorders identified in the included studies were major depressive disorder, substance use disorder, anxiety, and traumatic experiences. This highlights a research gap in TMHIs for other prevalent conditions in LMICs, like bipolar disorder, personality disorders, and schizophrenia (52, 53). Research has consistently confirmed the significant prevalence of these other mental health disorders in LMICs, yet they were not well represented in this review (5, 6). Addressing these gaps is crucial for developing relevant tele-mental health solutions that meet the diverse mental health needs of populations in LMICs.

Barriers and facilitators were identified for both service users and tele-mental health providers. The most prevalent barriers identified were related to the physical environment that limited opportunities for service users to use interventions. Physical barriers included a lack of personal communication devices, unreliable internet and phone connectivity, and limited access to electricity. These issues highlight the infrastructural challenges faced by service users in LMICs that limit the delivery of tele-mental healthcare (33, 54). Social barriers around privacy concerns and stigma which can interfere with the care received or deter individuals from seeking tele-mental health services were also noted. Additionally, barriers related to

capability for both service users and providers were identified. For patients, lack of information and literacy challenges impeded their ability to utilise tele-mental health services. When service users are not adequately informed about how tele-mental health works, its benefits, and how to navigate the technology involved, they may feel overwhelmed or confused, leading to reluctance or inability to seek help (33, 55). Moreover, low health literacy further complicates understanding of health-related information, making it difficult to follow instructions, complete forms, or communicate effectively with healthcare providers (42). Consequently, these factors contribute to barriers that prevent patients from fully utilising available TMHIs.

Results regarding facilitators showed that many mental healthcare providers held positive attitudes toward tele-mental health, which is also supported by several reviews (56-58). This positive attitude can be leveraged to address key issues related to limited knowledge and clear protocols for remote care to increase providers' psychological capability, especially their confidence during consultations (33, 59). This can mitigate challenges associated with the successful implementation of TMHIs (55, 60). Several facilitators were identified that can enhance the effective implementation of TMHIs. For instance, both patients and providers felt that tele-mental health improved access to care through cost savings and increased time efficiency. These advantages align with existing literature and are particularly pertinent in rural, remote and underserved areas where mental health services are limited (9, 55). Furthermore, the flexibility of tele-mental health allows patients to receive care without the need for long-distance travel, reducing the burden of other responsibilities, which is significantly beneficial in LMICs (22, 23). Additional facilitators were identified that can help overcome barriers related to psychological capability. For instance, providing telehealth-specific information on privacy, confidentiality, and stigma— can build confidence in service users and foster a safe, supportive environment that encourages utilisation of tele-mental health services (61). Such measures also influence the identified motivational facilitators by enhancing feelings of engagement and comfort with mental healthcare providers and willingness to use tele-mental health services if provided (59).

For providers, common key facilitators related to reflective motivation. Reported facilitators showed that mental healthcare providers had a positive attitude towards tele-mental health, recognising its benefits for patients, and confidence in delivering TMHIs, which is essential for successful implementation (58). Providers expressed high interest to receive training to improve their ability and confidence to deliver and develop TMHIs, thereby addressing barriers related to reflective motivation. Moreover, the ability to conduct more consultations due to reduced travel time was a critical opportunity and facilitator also linked to cost-saving from reduced travel expenses. Furthermore, identified capability facilitators demonstrated the adaptability of providers to transition to virtual care, enabling the delivery of a diverse range of TMHIs. This highlights the flexibility of providers and aligns with contemporary trends in mental healthcare, reflecting the growing acceptance and integration of TMHIs (58). These findings also revealed that the punctuality and consistency of mental healthcare providers facilitated a strong therapeutic alliance, which is crucial for achieving positive outcomes from the intervention (59). These facilitators highlight the potential of TMHIs to overcome some key barriers faced during mental healthcare delivery in LMICs.

Limitations of the review

The exclusion of grey literature and non-empirical evidence might have unintentionally omitted valuable insights, particularly from non-academic and MEDTECH sources that may report on practical implementations and challenges (62). Whilst efforts were made to include diverse geographical regions, the dominance of studies from South-East Asia, Africa and Eastern Mediterranean may limit generalisability of findings to other LMICs. Nonetheless, our review offers unique indications of factors affecting implementation of appropriate tele-mental healthcare in LMICs.

Implications for Practice and Policy

The COVID-19 pandemic spurred the pivot to digital and virtual healthcare, putting a bigger spotlight on concepts like Healthcare 4.0 and Society 5.0, that are about leveraging digital

technologies, data analytics, and Artificial Intelligence to revolutionise healthcare, solve socio-economic challenges and improve the quality of life (63). This scoping review is timely as it offers researchers and policymakers an early insight into the potential barriers and facilitators in the effective, fair and equitable implementation of tele-mental healthcare services for remote, rural, and underserved communities in LMICs. At chrono-policy levels, governments and policymakers need to consider the importance of a supportive and synchronised policy environment coupled with enabling incentives for careful planning and development of critical tele-mental health infrastructure that ensures privacy, connectivity and continuity. While acknowledging the inevitable competition for public funds, persuading governments and policymakers to prioritise infrastructure for tele-mental health is vital for building sustainable and resilient healthcare systems, ultimately leading to improved population well-being and long-term economic prosperity in LMICs.

Investing in stable and continuous electricity provision, potentially through renewable energy sources such as solar power, will ensure that both service users and healthcare providers can consistently access tele-mental health platforms (65). Establishing community telehealth centres equipped with reliable internet and digital devices could also provide a centralised location for patients to receive tele-mental health services (66). Moreover, fostering telehealth partnerships with local telecommunications companies and NGOs to subsidise internet data costs and provide affordable communication devices to communities in need is an essential strategy to enhance accessibility (67). Furthermore, partnering with local schools and nearby community hubs with reliable internet access and connectivity can facilitate implementation of TMHIs in areas with unreliable internet access and connectivity (68). Urban locations in LMICs generally exhibit greater access to digital technologies, characterised by higher rates of internet connectivity and a more widespread availability of personal communication devices (33). This disparity allows residents in urban areas to more readily utilise tele-mental health services compared to their counterparts in rural and remote regions (28, 69). Simple, low-bandwidth tele-mental health platforms that are designed to function effectively with limited

internet connectivity, such as text and audio communication instead of video, can be an access solution for rural, remote and underserved areas.

To address barriers related to social opportunity and psychological capability, the Behaviour Change Wheel framework emphasises the significance of educational initiatives (40). This may involve community-based awareness programs designed to increase awareness of the various tele-mental health services available, whilst providing information about security and confidentiality of their personal information (70). Community-based programs can significantly influence societal attitudes, thereby encouraging individuals to seek assistance (71, 72). For healthcare providers, educational initiatives should focus on enhancing knowledge concerning TMHIs. Furthermore, a variety of guidelines are available to assist in the implementation of tele-mental health services, including those provided by the American Psychological Association and the American Telemedicine Association (59). Development of local guidelines or the adaptation of existing international guidelines to suit local context and needs would provide crucial support for grassroots initiatives focused on developing and implementing tele-mental health services.

Lastly, to mitigate barriers related to reflective motivation, the Behaviour Change Wheel framework recommends focusing on training programs for healthcare providers aimed at developing competencies in tele-mental health delivery, to enhance confidence in using these services (40, 73). These programs should encompass effective communication strategies and proficient use of technology (74). Communication strategies must include providing patients with information regarding critical issues such as patient privacy, data security, and safety strategies. In this context, providers can reference various laws that protect patient privacy and safeguard medical information during both in-person and telehealth encounters (75). However, it is worth noting the complexity that comes with regulation of patient data and safety considering country specific and region specific variation of legislation. An added complexity is the potential different levels of trust regarding data protection across countries. These issues

contribute to reducing social opportunity barriers related to patient privacy and data security (40).

Furthermore, training local health workers to assist with tele-mental health consultations can facilitate the setup of technology, enhance communication between patients and remote mental health professionals, and ensure the smooth operation of sessions (63, 76). Addressing these factors can advance the integration of tele-mental health services with existing healthcare systems and promote a more equitable distribution of these services, ultimately improving healthcare outcomes for rural, remote and underserved populations.

Conclusions

This scoping review uniquely highlights the challenges specific to individuals living in remote, rural, and underserved areas in LMICs, filling a critical gap by identifying unique barriers and facilitators to implementation of TMHIs, and offering evidence to inform future interventions. It highlights the potential for tele-mental health to improve access to mental health services for these communities. While significant barriers exist in accessing TMHIs in LMICs, targeted strategies addressing infrastructural, educational, and training needs can enhance the effectiveness of these interventions. By leveraging the facilitators identified in this review, policymakers and healthcare providers can work towards more equitable and accessible mental healthcare systems.

Supporting information

Acknowledgements

This review was conducted as part of a dissertation project for a master's degree in mental health awarded to co-author, Aysha Groen at Maastricht University.

Funding

This work is supported by the Worldwide Universities Network – Research Development Fund 2023 awarded to Dr Bessie Malila. Lisa Ballard's time was funded/supported by the National

Institute for Health and Care Research (NIHR) Southampton Biomedical Research Centre. Bessie Malila and Sandra Jumbe's time was also funded through the African Research Initiative for Scientific Excellence (ARISE) pilot programme (Grant No. DCI-PANAF/2020/420-028) implemented by the African Academy of Sciences with support from the European Commission and the African Union Commission. The views expressed are those of the author(s) and not those of the listed funders.

Authors' contributions

SJ drafted the scoping review protocol and together with LB, SL and BM collectively contributed to the conception and design of the study. AG conducted the search strategy, while title and abstract screening were conducted by AG and DM in supervision from SJ. SJ led and managed the data extraction process and reference lists of selected studies which were conducted by AG, AT, DN and VS. AG conducted the data analysis. The initial manuscript drafting was carried out by AG and SJ. All other coauthors (LB, SL, AT, DN, VS, DM, RR, JPR, WWSM, DL & BM) reviewed and approved subsequent and the final manuscript versions.

Conflicts of interest

There is no conflict of interest in this project.

References

1. Bhugra D, Moussaoui D, Craig TJ. Oxford textbook of social psychiatry: Oxford University Press; 2022.
2. Javed A, Lee C, Zakaria H, Buenaventura RD, Cetkovich-Bakmas M, Duailibi K, et al. Reducing the stigma of mental health disorders with a focus on low- and middle-income countries. Asian Journal of Psychiatry. 2021;58:102601.
3. Rathod S, Pinninti N, Irfan M, Gorczynski P, Rathod P, Gega L, et al. Mental health service provision in low-and middle-income countries. Health services insights. 2017;10:1178632917694350.
4. Patel V. Mental health in low-and middle-income countries. British medical bulletin. 2007;81(1):81-96.
5. Lund C. Poverty and mental health: Towards a research agenda for low and middle-income countries. Commentary on Tampubolon and Hanandita (2014). Social Science & Medicine. 2014;111:134-6.
6. Osborn TL, Wasil AR, Weisz JR, Kleinman A, Ndetei DM. Where is the global in global mental health? A call for inclusive multicultural collaboration. General Psychiatry. 2020;33(6):e100351.
7. Kirkbride JB, Anglin DM, Colman I, Dykxhoorn J, Jones PB, Patalay P, et al. The social determinants of mental health and disorder: evidence, prevention and recommendations. World Psychiatry. 2024;23(1):58-90.
8. Nazemi M, Kiani S, Zakerabasali S. Tele-mental health during the COVID-19 pandemic: A systematic review of the literature focused on technical aspects and challenges. Health Science Reports. 2023;6(10):e1637.
9. Organisation WH. World mental health report: Transforming mental health for all: World Health Organization; 2022.
10. Esponda GM, Hartman S, Qureshi O, Sadler E, Cohen A, Kakuma R. Barriers and facilitators of mental health programmes in primary care in low-income and middle-income countries. The Lancet Psychiatry. 2020;7(1):78-92.
11. Alloh FT, Regmi P, Onche I, van Teijlingen E, Trenoweth S. Mental health in low-and middle income countries (LMICs): going beyond the need for funding. Health Prospect: Journal of Public Health. 2018;17(1):12-7.
12. Turner S, Isaac V, Lim D. A qualitative study of rural and remote Australian general practitioners' involvement in high-acuity patients. International Journal of Environmental Research and Public Health. 2023;20(5):4548.
13. Kola L, Kohrt BA, Hanlon C, Naslund JA, Sikander S, Balaji M, et al. COVID-19 mental health impact and responses in low-income and middle-income countries: reimagining global mental health. Lancet Psychiatry. 2021;8(6):535-50.
14. Gureje O, Oladeji BD. Quality care for people with severe mental disorders in low-resource settings. The Lancet Psychiatry. 2022;9(1):3-5.
15. Saxena S, Lora A, Morris J, Berrino A, Esparza P, Barrett T, et al. Focus on global mental health: mental health services in 42 low-and middle-income countries: a WHO-AIMS cross-national analysis. Psychiatric Services. 2011;62(2):123-5.
16. Mistry SK, Ali AM, Yadav UN, Das S, Akter N, Huda MN, et al. COVID-19 related anxiety and its associated factors: a cross-sectional study on older adults in Bangladesh. BMC Psychiatry. 2022;22(1):737.
17. Hayes K, Blashki G, Wiseman J, Burke S, Reifels L. Climate change and mental health: risks, impacts and priority actions. International journal of mental health systems. 2018;12:1-12.
18. Knapp M, Wong G. Economics and mental health: the current scenario. World Psychiatry. 2020;19(1):3-14.
19. Sporinova B, Manns B, Tonelli M, Hemmelgarn B, MacMaster F, Mitchell N, et al. Association of mental health disorders with health care utilization and costs among adults with chronic disease. JAMA network open. 2019;2(8):e199910-e.

20. Gajarawala SN, Pelkowski JN. Telehealth benefits and barriers. The Journal for Nurse Practitioners. 2021;17(2):218-21.
21. Acharibasam JW, Wynn R. Telemental health in low-and middle-income countries: A systematic review. International Journal of Telemedicine and Applications. 2018;2018(1):9602821.
22. Ibragimov K, Palma M, Keane G, Ousley J, Crowe M, Carreño C, et al. Shifting to tele-mental health in humanitarian and crisis settings: an evaluation of Médecins Sans Frontières experience during the COVID-19 pandemic. Conflict and health. 2022;16(1):6.
23. Naslund JA, Mitchell LM, Joshi U, Nagda D, Lu C. Economic evaluation and costs of telepsychiatry programmes: A systematic review. Journal of Telemedicine and Telecare. 2022;28(5):311-30.
24. McLean S, Sheikh A, Cresswell K, Nurmatov U, Mukherjee M, Hemmi A, et al. The impact of telehealthcare on the quality and safety of care: a systematic overview. PLoS One. 2013;8(8):e71238.
25. Pandya A, Shah K, Chauhan A, Saha S. Innovative mental health initiatives in India: A scope for strengthening primary healthcare services. Journal of Family Medicine and Primary Care. 2020;9(2):502-7.
26. Narasimhan M, Druss BG, Hockenberry JM, Royer J, Weiss P, Glick G, et al. Impact of a Telepsychiatry Program at Emergency Departments Statewide on the Quality, Utilization, and Costs of Mental Health Services. Psychiatric Services. 2015;66(11):1167-72.
27. Zhao X, Bhattacharjee S, Innes KE, LeMasters TJ, Dwibedi N, Sambamoorthi U. The impact of telemental health use on healthcare costs among commercially insured adults with mental health conditions. Current medical research and opinion. 2020;36(9):1541-8.
28. Adiukwu F, de Filippis R, Orsolini L, Gashi Bytyçi D, Shoib S, Ransing R, et al. Scaling up global mental health services during the COVID-19 pandemic and beyond. Psychiatric Services. 2022;73(2):231-4.
29. Langarizadeh M, Tabatabaei MS, Tavakol K, Naghipour M, Rostami A, Moghbeli F. Telemental Health Care, an Effective Alternative to Conventional Mental Care: a Systematic Review. Acta Inform Med. 2017;25(4):240-6.
30. Haun MW, Oeljeklaus L, Hoffmann M, Tönnies J, Wensing M, Szecsenyi J, et al. Primary care patients' experiences of video consultations for depression and anxiety: a qualitative interview study embedded in a randomized feasibility trial. BMC Health Services Research. 2023;23(1):9.
31. Zhou W, Yu Y, Yang M, Chen L, Xiao S. Policy development and challenges of global mental health: a systematic review of published studies of national-level mental health policies. BMC psychiatry. 2018;18:1-9.
32. Nadkarni A, Gandhi Y, Bhatia U, Velleman R. Closing the treatment gap for alcohol use disorders in low- and middle-income countries. Glob Ment Health (Camb). 2023;10:e3.
33. Chakrabarti S. Digital psychiatry in low-and-middle-income countries: new developments and the way forward. World Journal of Psychiatry. 2024;14(3):350.
34. Curioso WH. Building capacity and training for digital health: challenges and opportunities in Latin America. Journal of medical Internet research. 2019;21(12):e16513.
35. Tricco AC, Lillie E, Zarin W, O'Brien KK, Colquhoun H, Levac D, et al. PRISMA extension for scoping reviews (PRISMA-ScR): checklist and explanation. Annals of internal medicine. 2018;169(7):467-73.
36. Barros Leal Farias D. Unpacking the 'developing'country classification: origins and hierarchies. Review of International Political Economy. 2024;31(2):651-73.
37. Gupta A, Singh A, Aneja K, Aggarwal V, Wadhwa J, Abraham D. How to write a scoping review?–A comprehensive guide. Endodontology. 2023;35(1):9-14.
38. Ouzzani M, Hammady H, Fedorowicz Z, Elmagarmid A. Rayyan—a web and mobile app for systematic reviews. Systematic reviews. 2016;5:1-10.
39. Lisy K, Porritt K. Narrative Synthesis: Considerations and challenges. JBI Evidence Implementation. 2016;14(4):201.
40. Michie S, Atkins L, West R. The behaviour change wheel. A guide to designing interventions. 2014;1:1003-10.

41. Michie S, van Stralen MM, West R. The behaviour change wheel: a new method for characterising and designing behaviour change interventions. Implement Sci. 2011;6:42.
42. Bhat A, Goud BR, Pradeep J, Jayaram G, Radhakrishnan R, Srinivasan K. Can mobile health improve depression treatment access and adherence among rural Indian women? A qualitative study. Culture, Medicine, and Psychiatry. 2020;44:461-78.
43. Koly KN, Saba J, Muzaffar R, Modasser RB, M TH, Colon-Cabrera D, et al. Exploring the potential of delivering mental health care services using digital technologies in Bangladesh: A qualitative analysis. Internet Interv. 2022;29:100544.
44. Munthali-Mulemba S, Figge CJ, Metz K, Kane JC, Skavenski S, Mwenge M, et al. Experiences and Perceptions of Telephone-delivery of the Common Elements Treatment Approach for Mental Health Needs Among Young People in Zambia During the COVID-19 Pandemic. Front Public Health. 2022;10:906509.
45. Bhardwaj A, Subba P, Rai S, Bhat C, Ghimire R, Jordans MJ, et al. Lessons learned through piloting a community-based SMS referral system for common mental health disorders used by female community health volunteers in rural Nepal. BMC Research Notes. 2020;13(1):309.
46. Clough BA, Zarean M, Ruane I, Mateo NJ, Aliyeva TA, Casey LM. Going global: do consumer preferences, attitudes, and barriers to using e-mental health services differ across countries? Journal of Mental Health. 2019;28(1):17-25.
47. Estapé T, Phillip EJ, Bergerot CD. Use of telehealth and eHealth technologies in patient care by psycho-oncology service providers in low–middle-income countries during the COVID-19. Psycho-oncology. 2021;31(2):334.
48. Knaevelsrud C, Brand J, Lange A, Ruwaard J, Wagner B. Web-based psychotherapy for posttraumatic stress disorder in war-traumatized Arab patients: randomized controlled trial. J Med Internet Res. 2015;17(3):e71.
49. Ganesh R, Verma R, Deb KS, Chadda RK. Learning from telepsychiatry during COVID-19 pandemic in India: Boon for public mental health in low-& middle-income countries. Indian Journal of Medical Research. 2022;155(1):197-9.
50. Gruber J, Mendle J, Lindquist KA, Schmader T, Clark LA, Bliss-Moreau E, et al. The future of women in psychological science. Perspectives on Psychological Science. 2021;16(3):483-516.
51. Wendt D, Shafer K. Gender and attitudes about mental health help seeking: results from national data. Health & social work. 2016;41(1):e20-e8.
52. Arnbjerg C, Rurangwa NU, Musoni-Rwililiza E, Gishoma D, Carlsson J, Kallestrup P. Intervention trials for adults with bipolar disorder in low-income and lower-middle-income countries: A systematic review. Journal of Affective Disorders. 2022;311:256-66.
53. Asher L, Fekadu A, Hanlon C. Global mental health and schizophrenia. Current opinion in psychiatry. 2018;31(3):193-9.
54. Jha AK, Sawka E, Tiwari B, Dong H, Oh CC, Ghaemi S, et al. Telemedicine and Community Health Projects in Asia. Dermatol Clin. 2021;39(1):23-32.
55. Weightman M. Digital psychotherapy as an effective and timely treatment option for depression and anxiety disorders: Implications for rural and remote practice. Journal of international medical research. 2020;48(6):0300060520928686.
56. Fouad AA, Osman MA, Abdelmonaem YMM, Karim NAHA. Awareness, knowledge, attitude, and skills of telemedicine among mental healthcare providers. Middle East Current Psychiatry. 2023;30(1):5.
57. Guinart D, Marcy P, Hauser M, Dwyer M, Kane JM. Mental health care providers' attitudes toward telepsychiatry: a systemwide, multisite survey during the COVID-19 pandemic. Psychiatric Services. 2021;72(6):704-7.
58. Connolly SL, Miller CJ, Lindsay JA, Bauer MS. A systematic review of providers' attitudes toward telemental health via videoconferencing. Clinical Psychology: Science and Practice. 2020;27(2):e12311.
59. Palmer CS, Levey SMB, Kostiuk M, Zisner AR, Tolle LW, Richey RM, et al. Virtual care for behavioral health conditions. Primary care. 2022;49(4):641.

60. Combi C, Pozzani G, Pozzi G. Telemedicine for developing countries. Applied clinical informatics. 2016;7(04):1025-50.
61. Bolton P, West J, Whitney C, Jordans MJ, Bass J, Thornicroft G, et al. Expanding mental health services in low-and middle-income countries: A task-shifting framework for delivery of comprehensive, collaborative, and community-based care. Cambridge Prisms: Global Mental Health. 2023;10:e16.
62. Sharma H, Verma S. Is positive publication bias really a bias, or an intentionally created discrimination toward negative results? Saudi Journal of Anaesthesia. 2019;13(4):352-5.
63. Arnold AC, Fleet R, Lim D. A case for mandatory ultrasound training for rural general practitioners: a commentary. Rural Remote Health. 2021;21(3):6328.
64. Shaengchart Y, Kraiwanit T. Starlink satellite project impact on the Internet provider service in emerging economies. Research in Globalization. 2023;6:100132.
65. Hadwan M, Alkholidi A. Solar power energy solutions for Yemeni rural villages and desert communities. Renewable and Sustainable Energy Reviews. 2016;57:838-49.
66. Shin P, Sharac J, Jacobs F. Provision of telemedicine services by community health centers. Online journal of public health informatics. 2014;6(2):e185.
67. Murillo MJ, Paco JA, Wright D. Long-Distance Telecommunication in Remote Poor Areas: From Partnerships and Implementation to Sustainability. IEEE Technology and Society Magazine. 2015;34(1):19-30.
68. Marcin JP, Shaikh U, Steinhorn RH. Addressing health disparities in rural communities using telehealth. Pediatr Res. 2016;79(1-2):169-76.
69. Ojeahere MI, Kiburi SK, Agbo P, Kumar R, Jaguga F. Telehealth interventions for substance use disorders in low-and-middle income countries: A scoping review. PLOS Digital Health. 2022;1(11):e0000125.
70. Semrau M, Gronholm PC, Eaton J, Maulik PK, Ayele B, Bakolis I, et al. Reducing stigma and improving access to care for people with mental health conditions in the community: protocol for a multi-site feasibility intervention study (Indigo-Local). International Journal of Mental Health Systems. 2024;18(1):35.
71. Mabele OM, Benedict MOA, Steinberg WJ, Reji E, Van Rooyen C, Adefuye AO. Knowledge, attitudes and practices of men in a South African rural community in relation to exclusive breastfeeding. S Afr Fam Pract (2004). 2022;64(1):e1-e8.
72. Jumbe S, Nyali J, Simbeye M, Zakeyu N, Motshewa G, Pulapa SR. 'We do not talk about it': Engaging youth in Malawi to inform adaptation of a mental health literacy intervention. PloS one. 2022;17(3):e0265530.
73. Gibson C, Smith D, Morrison A. Improving health literacy knowledge, behaviors, and confidence with interactive training. HLRP: Health Literacy Research and Practice, 6 (2), e113–e120. 2022.
74. Schroeder S, Roberts H, Heitkamp T, Clarke B, Gotham HJ, Franta E. Rural mental health care during a global health pandemic: Addressing and supporting the rapid transition to tele-mental health. Journal of Rural Mental Health. 2021;45(1):1.
75. Balestra M. Telehealth and legal implications for nurse practitioners. The Journal for Nurse Practitioners. 2018;14(1):33-9.
76. Ralston AL, Holt NR, Hope DA. Tele-mental health with marginalized communities in rural locales: Trainee and supervisor perspectives. Journal of Rural Mental Health. 2020;44(4):268.

Figure 1 PRISMA

Click here to access/download;Figure;WUN SR PRISMA diagram 10.4.25.docx

Figure 1: PRISMA flow diagram

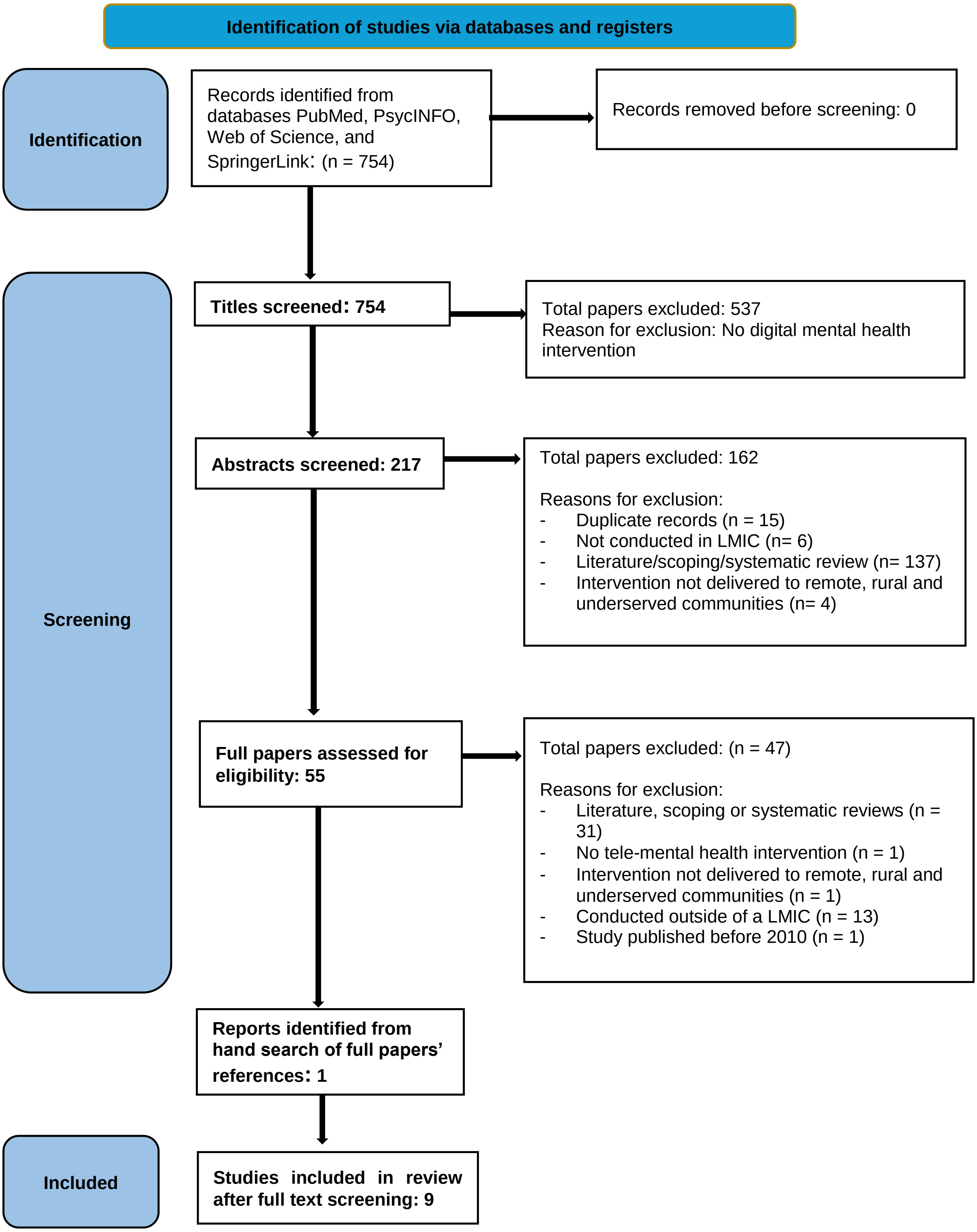

Click here to access/download
Supporting Information
WUN PRISMA-ScR Checklist 18.6.25.docx

Supplementary appendices

Click here to access/download

Supporting Information

WUN scoping review supplementary file 18.6.25.docx
