# Supplementary material for: A scoping review of barriers and facilitators to implementing tele-mental health solutions for rural, remote and underserved populations in low- and middle-income countries
Source: PLOS Digit Health. 2026 Jun 25;5(6):e0000903. doi: 10.1371/journal.pdig.0000903 (PMC13298784; doi:10.1371/journal.pdig.0000903)
Supplement: Attachment — Submitted filename: PLOS Digital Health responses to reviewer comments 14.10.25.docx [file pdig.0000903.s006.docx]

To: Dr Leo Anthony Celi

Editor in Chief

PLOS Digital Health

14^th^ October 2025

Dear Dr Celi & the editorial team

**RE: Manuscript ID PDIG-D-25-00312**

On behalf of my colleagues, I thank you for the opportunity to revise our manuscript titled *"A scoping review of barriers and facilitators to implementing tele-mental health solutions for rural, remote and underserved populations in low- and middle-income countries"* which we submitted to your journal, PLOS Digital Health in May 2025. We note the comments, thank you. Please see below our responses to the three reviewers’ comments received.

**Reviewer 1 comments**

General Comments: The manuscript entitled “A Scoping Review of Barriers and Facilitators to Implementing Tele-Mental Health Solutions for Rural, Remote and Underserved Populations in Low- and Middle-Income Countries” addresses a highly relevant and timely topic in global mental health. It targets an evident research gap concerning the deployment of digital mental health interventions in low-resource settings. The manuscript is generally well-structured and clearly written. The methodological approach, especially the use of the COM-B model, is appropriate and provides a valuable framework for categorising barriers and facilitators.
However, certain aspects of the study could be further strengthened. For example, providing more detail regarding the limitations of the search strategy, elaborating on the contextual nuances of the LMIC definition, and offering deeper discussion on gender dynamics or broader social inequities would enhance the manuscript’s contribution. While the language is mostly clear and academic, several sections could benefit from careful language polishing to improve precision and readability. Overall, I consider this a promising and relevant manuscript that can make a significant contribution to the field of digital health, provided the authors address the points raised in this review.

Response to reviewer: Thank you for acknowledging the high relevance and timely nature of our work. Your comments are well received, and we have revised our manuscript to address the gaps you have noted.

**1. Breakdown of reviewer 1 major comments**

***Reviewer 1 comment 1:*** The manuscript focuses on LMICs; however, the term “low- and middle-income countries” encompasses highly heterogeneous contexts in terms of economy, culture, and health systems. I suggest that the authors discuss in greater depth which regions are represented in the included studies and to what extent the findings can be generalized to LMICs as a whole.

***Response to reviewer 1 comments 1:*** This has been added to the discussion (page 22 lines 426-433). Also, per Reviewer 2 comments, the revision has reference to how LMICs are defined on page 3 lines 56-58

***Reviewer 1 comment 2.*** While the search strategy is clearly presented, it raises the question of whether sufficient databases were included, particularly those specific to LMIC contexts (e.g., LILACS, AJOL, or regional databases). I recommend that the authors justify their choice of databases and discuss what types of studies might have been missed due to this limitation.

***Response to reviewer 1 comment 2:*** Thank you. We have added a justification for the databases chosen to the methods section (page 8 lines 165-171) and reflections on the limitations (page 26 lines 510-514)

***Reviewer 1 comment 3.*** The manuscript briefly mentions sociodemographic factors, but the discussion of gender-related issues and broader social inequalities remains superficial. I encourage the authors to elaborate on how these factors impact access to tele-mental health services in LMIC settings.
***Response to reviewer 1 comment 3***: This has been added to the discussion (page 23 lines 437-442)

***Reviewer 1 comment 4***. The limitations section is relatively brief. I recommend that the authors expand on potential biases, such as publication bias, language bias (given that only English-language studies were included), and any limitations of the COM-B model when applied across diverse cultural contexts.
***Response to reviewer 1 comment 4:*** This has been added to the discussion (page 26 lines 507-522)

***Reviewer 1 comment 5***: While the use of the COM-B model is an excellent choice, it remains unclear how exactly the categorisation was conducted. For example, how many researchers performed the coding, and how was interrater reliability ensured? I suggest that the authors provide more details about the coding process.
***Response to reviewer 1 comment 5:*** This detail has now been added to the methods section (page 14 lines 223-233).

***Reviewer 1 comment 6:*** The study references various tele-mental health interventions (e.g., telephone counselling, video consultations, mobile apps), but it remains unclear which barriers or facilitators are more strongly associated with each type of intervention. I suggest that the authors provide a more nuanced discussion distinguishing which challenges pertain to specific service modalities.
***Response to reviewer 1 comment 6***: We have not provided a detailed discussion of which barriers or facilitators are more strongly linked to implementation of specific service modalities/ telemental health interventions. This is because of several reasons. Firstly, the nine included studies were highly heterogeneous in terms of study design, population, setting, and type of telemental health solution, making it difficult to draw meaningful comparisons about the relative strength of individual barriers or facilitators. Secondly, most studies provided narrative or descriptive accounts rather than quantitative measures of intervention effect, so assessing the relative influence of specific factors on implementation risks overinterpretation of limited or context-specific data. Thirdly, the poor description of interventions as compared to TIDieR does not permit us to distil information at the necessary level. However, we do highlight observable patterns where certain barriers (e.g., lack of non-verbal cues) were more commonly associated with audio-only platforms (lines 324-327), and others (e.g., privacy concerns) spanned across modalities (lines 459-460). Future work could include a realist review on which barriers or facilitators are more strongly associated with each type of intervention if empirical data permits.

***Reviewer 1 comment 7***: While the manuscript’s conclusion is valuable, it primarily contains general statements. I encourage the authors to develop more concrete practical recommendations (e.g., implementation strategies, policy suggestions) for decision-makers and practitioners, particularly relevant for LMIC contexts.

***Response to reviewer 1 comment 7:*** Noted. The conclusion has been edited to reflect practical recommendations (lines 602 -619)


**2. Breakdown of reviewer 1 minor comments**

***Reviewer 1 minor comment 1***. Abstract. Replace “poses a great global public health concern” with the more academic phrasing: “represents a significant global public health concern.”
***Response to reviewer 1 comment 1:*** Edit suggestions done (line 28)

***Reviewer 1 minor comment 2***: Abstract. Consider simplifying and splitting this long sentence: “Tele-mental health, which involves remote access to care through information and communications technologies (ICTs) and mobile communication systems, may represent a promising solution.”
***Response to reviewer 1 comment 2***: Edit suggestions done (lines 30-31)

***Reviewer 1 minor comment 3:*** Introduction. Insert hyphen in compound number: “Eighty-two percent of the global population struggling with at least one diagnosable health condition live in LMICs.”
***Response to reviewer 1 comment 3:*** Edit suggestions done (line 60)

***Reviewer 1 minor comment 4:***. Introduction. In standard academic writing, “social determinants of health” is usually lowercase unless referring to a specific framework. Change to: “social determinants of health such as low income, low education, and social exclusion are key contributors to mental health challenges.”
***Response to reviewer 1 comment 4:*** Edit suggestions done (line 62)

***Reviewer 1 minor comment 5:*** Introduction. “Access to appropriate mental health care remains a challenge in LMICs for various reasons.” Here, consider the more precise academic phrase: “continues to be a significant challenge in LMICs for various reasons.”
***Response to reviewer 1 comment 5***: Edit suggestions done (line 70)

***Reviewer 1 minor comment 6:*** Methods. “Publications in English, Dutch, Cantonese Chinese, Spanish, and French were considered as our review team comprised individuals from diverse backgrounds who collectively spoke these languages.” Split this long sentence for clarity: “Publications in English, Dutch, Cantonese Chinese, Spanish, and French were considered. Our review team comprised individuals from diverse backgrounds who collectively spoke these languages.”
***Response to reviewer 1 comment 6***: Edit suggestions done (line 149)

***Reviewer 1 minor comment 7:*** Discussion. “emerges as a promising alternative” could be more formal: “is positioned as a potentially effective alternative.”

***Response to reviewer 1 comment 7***: Edit suggestions done (line 409)

**Reviewer 2 comments**

***Reviewer 2 general comment:*** Thank you for the opportunity to review this well-written article. This is a valuable piece of work which reviews the barriers and facilitators (for both service users and service providers) to online mental health support in low- and middle-income countries (LMICs). The barriers and facilitators identified are specific to their contexts in LMICs, as are the recommendations for future practice and policy. This detailed review will be useful for policymakers and future researchers in this field, so I recommend acceptance of this article, with some very minor revisions suggested based on the feedback below.
***Response to reviewer 2 general comment:***- Thank you for noting the importance of our work

***Reviewer 2 comment 1:*** The introduction should include a definition of LMICs, especially for an audience interested in Digital Health who may not be familiar with this terminology. The World Bank definition is mentioned in the inclusion criteria, but not explained. This could be defined at the outset.
***Response to reviewer 2 comment 1:*** noted and now added (page 3 lines 57-59)

***Reviewer 2 comment 2:*** Publications were considered in English, Dutch, Cantonese Chinese, Spanish, and French. Even though all included publications were in English, it is a strength of the study that the authors were able to consider a wide range of publications.
***Response to reviewer 2 comment 2:***- thank you

***Reviewer 2 comment 3:*** The inclusion criteria lists “Remote, rural and/or underserved communities”, but it is not clear how “underserved communities” were defined. Further clarity here would be appreciated.
***Response to reviewer 2 comment 3:*** We previously defined this in the background of the original submitted manuscript (lines 120). Based on your feedback we have added more detail for added clarity (lines 125-128).

***Reviewer 2 comment 4:*** The text states that 147 studies were excluded after abstract screening and 47 excluded after full-text screening. The reasons for exclusion are not stated in-text, but they are stated in Figure 1. Signposting this to the reader would be helpful.
***Response to reviewer 2 comment 4:*** This has now been done (lines 179-181)

***Reviewer 2 comment 5:*** The results section is clear and well-structured. The discussion section summarises the findings well and links to broader issues, such as the prevalence of other mental health issues like bipolar disorder, but a lack of TMHIs for these.

***Response to reviewer 2 comment 5:*** Thank you

***Reviewer 2 comment 6:*** The implications for practice and policy section introduces concepts which need to be explained better for the reader. Healthcare 4.0 and Society 5.0 are referred to but should be explained and referenced. The term “chrono-policy levels” is introduced but it is not clear what this means. Finally, “NGO” should be spelled out instead of using an acronym.

***Response to reviewer 2 comment 6:*** This section is now revised to reflect your comments (page 26 lines 525-537)

**Reviewer 3 comments**

***General comments:*** I enjoyed reading your draft because of how nicely clearly written and how timely your research question is. Thanks very much for taking the time to do this research, it is meaningful.

***Reviewer 3 comment 1:*** Originality - High importance and broad interest to community of researchers, engineers and clinicians working in the field of digital health

***Response to reviewer 3 comment 1:*** Thank you for noting the importance of our work

***Reviewer 3 comment 2:*** Methods: moderate methodological rigor and ethical standards. I think you should have reviewed the abstracts by skimming to confirm that excluded studies were accurately excluded. It is surprising that only 9 studies were found although the geographical areas are broad. I think once you address this part of your review, then you will jump to high methodological rigor. This is the main reason I chose "Major revision" in my assessment because study titles do not always tell about research questions.

***Response to reviewer 3 comment 2:*** We did read through the abstracts of the 537 titles to confirm that they do not have TMHIs, ensuring accuracy of excluded studies.

***Reviewer 3 comment 3:*** Substantial evidence for its conclusions - I think this is moderate too, maybe you can discuss more literature on the digital health disparities in these regions or the lack of access to traditional health services so that you lay the ground for more robust conclusions beyond the 9 studies. and also if you include a "future directions" paragraph that has some evidence-based recommendations published in other studies in other regions that would further strengthen your paper.

***Response to reviewer 3 comment 3:*** Noted. We have revised the conclusion (lines 600-619) to include “future directions" within paragraph as based on evidence-based recommendations from published studies we have added to discussion following reviewers’ comments as well as findings from our WUN collaborative project case studies that will be published in the near future.

***Reviewer 3 comment 4:*** Clearly outlined utility and accessibility for the broader community - I think this was done ok overall, but if you integrate the above feedback on methods and substantial evidence for its conclusions section, then you will get better in this section as well.

***Response to reviewer 3 comment 4:*** As we have addressed your comments and those from the two other reviewers, we hope you feel this section is now better

***Reviewer 3 comment 5:*** Follow appropriate standards and practice of open science - I think you did a good job and writing an easily replicable study, but there was no link to the original database list of studies for people to take a look. You have included a search prompt only, which is good, but not enough to have this fully open science.

***Response to reviewer 3 comment 5:*** Noted. We will share a link of our review data from Rayyan as part of the manuscript submission. We will also load our data onto Open Science as part of wrapping up the work as an alternative to sharing raw data. Furthermore, details of our included studies are listed in table 3 for other researchers to look up and review.

Thank you for the reviewers’ insights and comments. We believe that their feedback has improved our manuscript.

Kind regards

Dr Jumbe
