# Supplementary material for: A scoping review of barriers and facilitators to implementing tele-mental health solutions for rural, remote and underserved populations in low- and middle-income countries
Source: PLOS Digit Health. 2026 Jun 25;5(6):e0000903. doi: 10.1371/journal.pdig.0000903 (PMC13298784; doi:10.1371/journal.pdig.0000903)
Supplement: Attachment — Submitted filename: PLOS Digital Health responses to reviewer comments 10.12.25.docx [file pdig.0000903.s008.docx]

To: Dr Leo Anthony Celi

Editor in Chief

PLOS Digital Health

17^th^ December 2025

Dear Dr Celi & the editorial team

**RE: Manuscript ID PDIG-D-25-00312R1**

On behalf of my colleagues, I thank you for a second opportunity to revise our manuscript titled *"A scoping review of barriers and facilitators to implementing tele-mental health solutions for rural, remote and underserved populations in low- and middle-income countries"* which we submitted to your journal, PLOS Digital Health in October 2025 after initial reviewers’ comments.

Please see below our responses to the three reviewers’ comments received.

**Reviewer 1 comments**

***Reviewer 1 general comment:*** The revised manuscript demonstrates substantial improvement and reflects the authors’ thorough engagement with the reviewers’ feedback. The inclusion of detailed methodological clarifications (e.g., coding process, COM-B model application) and the expanded discussion on social and gender inequities have significantly enhanced both transparency and depth. Conceptual clarifications, such as the definition of LMICs and “underserved” communities, further strengthen the paper’s precision and readability. Overall, the manuscript represents a valuable contribution to understanding global inequities in tele-mental health implementation and offers practical insights for policy and practice. I have only two additional major recommendations before final acceptance, both intended to enhance coherence and policy relevance.

***Response to reviewer 1 general comment:*** We thank you for your thoughtful evaluation and for recognising the improvements in methodological transparency, conceptual clarity, and discussion of social and gender inequities. We appreciate the additional recommendations to enhance coherence and policy relevance and have carefully addressed them in the revised manuscript.

***Reviewer 1 comment 1:*** While the revised manuscript offers a clear regional overview, it would benefit from a brief integrative synthesis that identifies which barriers and facilitators appear universal across LMICs and which are region-specific. Adding a concise paragraph to the end of the Discussion or Implications for Policy section could strengthen cross-context comparability and enhance the paper’s relevance for global health decision-makers.

***Response to reviewer 1 comment 1:*** Thank you. We agree that an integrative synthesis noting universal and region-specific factors would substantially strengthen the comparative value and relevance of our findings for global policy. We have added a new paragraph to the Implications for Practice and Policy section to explicitly address this point (lines 542-558).

***Reviewer 1 comment 2:*** The conclusions outline meaningful future directions but remain somewhat general. I recommend specifying which types of research approaches (e.g., realist reviews, participatory implementation studies, mixed-methods longitudinal designs) or partnership models (e.g., NGO–academic collaborations) could most effectively advance sustainable tele-mental health implementation in LMICs. Such specification would enhance the practical value of the manuscript and provide clearer guidance for future research.

***Response to reviewer 1 comment 2:*** We appreciate this suggestion; specifying how future work should be conducted will significantly enhance the practical value of our conclusions. We have substantially strengthened the 'Future Research' paragraph (lines 659-684) within the 'Conclusion and future directions' section. We have incorporated specific methodological recommendations (e.g., mixed-methods designs) and explicitly included concrete partnership models (e.g., NGO-Telecommunications company alliances) to provide clear, strategic guidance for advancing equitable tele-mental health implementation in LMICs.

**Reviewer 2 comments**

***Reviewer 2 general comment:*** Thank you for the opportunity to review the revised manuscript. All my previous comments have been addressed.

***Response to reviewer 2 general comment:*** Thank you for your careful review of our revised manuscript. We appreciate your positive feedback and are pleased that our revisions have addressed all your comments.

**Reviewer 3 comments**

***General comments:*** Thank you very much for addressing many of my comments and feedback. You missed many of my comments on the attached pdf I have provided and here they are again as a response to your response.

***Response to reviewer 3 general comment:*** Thank you for your continued review and for providing your comments again. We apologise for any oversights and have carefully addressed all points from the PDF document you shared within this revised manuscript. Please see our detailed responses below.

***Reviewer 3 comment 1:*** Line 148 Table 2: Search strategies used within the scoping review : you did not address my comment on the definition of mental health intervention. A mental health intervention could be for a physical condition like behavioral medicine services, was that included in the search or excluded? That should be clarified.
***Response to reviewer 3 comment 1:*** Comment noted. As stated in our background, our scoping review aimed to explore barriers and facilitators to implementing telemental health solutions in underserved communities within LMICs to inform digital strategies that can specifically improve access to quality and affordable mental healthcare. Within this scope, a mental health intervention was ‘any action, strategy, or support system designed to help someone improve their mental health, manage a mental disorder, or prevent symptoms of mental illness from worsening’. For example, studies with a digital intervention addressing depression in cancer patients from an underserved community would have been included in this review but a study exploring a digital intervention for cancer patients to improve treatment adherence (behavioural) would have been excluded as it is not primarily addressing mental health but behaviour change. We hope this is now clear.

We have now added a definition of ‘mental health intervention’ to the search strategy section of the manuscript (lines 145-148).

***Reviewer 3 comment 2:*** Line 150 Screening and selection of evidence - I suggested spilling out the names of reviewers so that the reader knows what the letters stand for, is this someone external or representing the names of authors basically.

***Response to reviewer 3 comment 2:*** Thank you for this suggestion. The letters represent study authors. We have now clarified the names of the reviewers in the “Screening and Selection of Evidence” section (e.g. lines 175, 190, etc) making this information transparent for the reader.

***Reviewer 3 comment 3:*** Line 306 and 307 not sure you fixed the redundancy in phrases I noted in my comments?
***Response to reviewer 3 comment 3:*** Yes. Duplication of this phrase ‘*list of permitted drugs for online sessions from Bangladesh College of Physicians and Surgeons’* on lines 342-343 has been removed. Thank you for pointing this out

***Reviewer 3 comment 4:*** Line 443 the phrase “For providers, common key facilitators related to reflective motivation.” is very confusing thrown in there without clear connection to context or paragraph, I suggest rewriting it.
***Response to reviewer 3 comment 4:*** noted. This has now been rewritten for clarity (lines 493-495)

***Reviewer 3 comment 5:*** Line 459 the limitations section: you did not address my comment on the pdf about defining the full spectrum of what is considered mental health interventions and whether you searched for lifestyle psychological interventions (i.e. behavioral medicine interventions) or not? This distinction is very important and it has to be described in the limitations section if you did not include it in the search which could lead to excluding unintentionally studies that could be fitting the criteria.
***Response to reviewer 3 comment 5:*** We thank you for this important point. As outlined in the background, our scoping review focused specifically on telemental health interventions aimed at improving access to quality and affordable mental healthcare. Within this scope, a telemental health intervention was “any action, strategy, or support system designed to help someone improve their mental health, manage a mental disorder, or prevent symptoms of mental illness from worsening” provided remotely/virtually/ digitally. Broader behavioural interventions, such as lifestyle interventions not primarily addressing mental health, were considered out of scope. We have not included this distinction in the Limitations section, as such interventions were not relevant for our review from the outset.

***Reviewer 3 comment 6:*** Line 475 I also have a comment on the pdf about the word “At chrono-policy levels” is not clear for me as a scholar and how about the general reader. Please define these terms in a glossary if not inside the text of the paragraph. Accessibility of terminology and jargon free papers are important for open science and accessibility of knowledge.
***Response to reviewer 3 comment 6:*** Thank you. We did address this comment in the previous manuscript revision by adding a brief explanation of what chrono-policy level means i.e. policies concerned with how changes over time affect an individual's interaction with their environment. We have now added a more detailed explanation in the paragraph (lines 560 to 564).

***Reviewer 3 comment 7:*** Line 479 I also commented on the pdf about adding some info about the economic burden of mental health problems and the lack of access in these communities so that your statement is more persuasive and effective.
***Response to reviewer 3 comment 7:***  Noted with thanks. This has been added to the Discussion within the policy and implications section (lines 569-574)

***Reviewer 3 comment 8:*** Line 511 I commented on the pdf about adding the WHO guidelines or policies on digital health which is also very relevant here and it is more persuasive to add a global health agency’s publication rather than a state-specific only like the US’s or the UK’s or Canada’s policies.
***Response to reviewer 3 comment 8:*** Thank you for this helpful suggestion. We agree that referencing World Health Organization (WHO) guidance strengthens the global relevance of the manuscript. We have revised the Implications for Practice and Policy section to explicitly reference the WHO Guideline on Digital Interventions for Health System Strengthening and the Global Strategy on Digital Health 2020–2027, alongside noting the need for local adaptation (lines 602-613). This revision shifts the emphasis from country-specific guidelines to globally applicable frameworks more relevant to LMIC contexts.

***Reviewer 3 comment 9:*** Line 468 Implications for Practice and Policy : you did not add any of the future questions that should be answered in future studies in order to overcome the limitations in yours (AKA the things you have missed in this study and you hopefully described in the limitations section, now you have to mention the need for studying or addressing these limitations in future studies.
***Response to reviewer 3 comment 9:*** thank you for this suggestion. This has now been added to the ‘Conclusion and future directions’ section of the manuscript (lines 659-684)

***Reviewer 3 comment 10:*** Finally, you have indicated in your response to my comment 3 as you see below in the paragraph that there were additions to the conclusion or discussion or future implications sections, but I couldn’t find anything about integrating the information I asked for in my comment in any of your sections nor the lines you have indicated except for citations. Are you missing something here? Or you have added only citations? It should be discussed in the sections described clearly, not only in citations.
Here is your response that I am referring to: “Response to reviewer 3 comment 3: Noted. We have revised the conclusion (lines 600-619) to include “future directions" within paragraph as based on evidence-based recommendations from published studies we have added to discussion following reviewers’ comments as well as findings from our WUN collaborative project case studies that will be published in the near future.”

***Response to reviewer 3 comment 10: address this original comment –*** Thank you for drawing our attention to this important point. We acknowledge that our earlier response did not clearly demonstrate how the requested information had been incorporated into the manuscript. We apologise for this lack of clarity. We agree that merely adding citations is insufficient and that the requested content must be explicitly discussed within the relevant sections.

We have revised the Discussion, Implications for Practice and Policy, and Conclusions and Future Directions sections to explicitly integrate broader literature on digital health disparities and limited access to traditional mental health services in LMICs. In the Discussion, we now clearly describe structural and infrastructural barriers (e.g., limited access to devices, unreliable connectivity, electricity constraints) in lines 457-460, social and gender-based inequities (lines 438-445), and systemic treatment gaps that disproportionately affect rural, remote, and underserved populations (lines 542-557). This evidence provides the conceptual and empirical foundation for the evidence-based recommendations articulated in the Conclusions and Future Directions section, including priorities for infrastructure investment, policy alignment, and context-sensitive implementation of tele-mental health interventions (lines 649-684).

Once again, we are very thankful of the reviewers’ continuous feedback and insights.

Yours sincerely

Dr Sandra Jumbe
