# Supplementary material for: A scoping review of barriers and facilitators to implementing tele-mental health solutions for rural, remote and underserved populations in low- and middle-income countries
Source: PLOS Digit Health. 2026 Jun 25;5(6):e0000903. doi: 10.1371/journal.pdig.0000903 (PMC13298784; doi:10.1371/journal.pdig.0000903)
Supplement: Attachment — Submitted filename: PLOS Digital Health responses to reviewer comments 6.3.26.docx [file pdig.0000903.s009.docx]

To: Dr Leo Anthony Celi

Editor in Chief

PLOS Digital Health

6^th^ March 2026

Dear Dr Celi & the editorial team

**RE: Manuscript ID PDIG-D-25-00312R2**

On behalf of my colleagues, I thank you for a third opportunity to make a minor revision to our manuscript titled *"A scoping review of barriers and facilitators to implementing tele-mental health solutions for rural, remote and underserved populations in low- and middle-income countries"* which we submitted to your journal, PLOS Digital Health in December 2025 after addressing the second round of reviewers’ comments.

Please see below our responses to the one outstanding comment received from reviewer 3.

**Reviewer 3 comment:** thanks for addressing my comments, there is one more. You will see it in the attached pdf on page 8, response to comment 5 of reviewer 3. I still think it would be better to mention the distinction between mental health intervention versus behavioral medicine intervention in the eligibility criteria section because the paper should be clear and transparent on its eligibility criteria and you don't want to mislead readers that those are included. transparency is good for you and for facilitating future directions based on the limitations of this study. In your response, you explained to me but did not explain that to the reader in the manuscript.

**Response to reviewer 3’s comment:** Thank you for this helpful suggestion. We agree that clarifying the distinction between tele-mental health interventions and broader behavioural medicine interventions improves the transparency of the eligibility criteria. We have now added statements in the inclusion and exclusion criteria section (see Table 1), to make clear that studies evaluating digital or telehealth interventions focused primarily on behavioural medicine (e.g., lifestyle modification or chronic disease self-management without a primary mental health focus) were excluded (page 7, line 154). The paragraph before Table 1 already has a definition of what we meant by a ‘mental health intervention’ for additional clarity (lines 145-148).

Once again, we are very thankful of the reviewers’ continuous feedback and insights.

Yours sincerely

Dr Sandra Jumbe
