# Supplementary material for: A scoping review of barriers and facilitators to implementing tele-mental health solutions for rural, remote and underserved populations in low- and middle-income countries
Source: PLOS Digit Health. 2026 Jun 25;5(6):e0000903. doi: 10.1371/journal.pdig.0000903 (PMC13298784; doi:10.1371/journal.pdig.0000903)
Supplement: Peer review history

# Peer review: Decision Letter 0

**DOI:** 10.1371/journal.pdig.0000903.r001

**Authors:** Haleh Ayatollahi

8 Aug 2025

PDIG-D-25-00312

A scoping review of barriers and facilitators to implementing tele-mental health solutions for rural, remote and underserved populations in low- and middle-income countries

PLOS Digital Health

Dear Dr. Jumbe,

Thank you for submitting your manuscript to PLOS Digital Health. After careful consideration, we feel that it has merit but does not fully meet PLOS Digital Health's publication criteria as it currently stands. Therefore, we invite you to submit a revised version of the manuscript that addresses the points raised during the review process.

Please submit your revised manuscript within 60 days Oct 07 2025 11:59PM. If you will need more time than this to complete your revisions, please reply to this message or contact the journal office at digitalhealth@plos.org. When you're ready to submit your revision, log on to https://www.editorialmanager.com/pdig/ and select the 'Submissions Needing Revision' folder to locate your manuscript file.

Please include the following items when submitting your revised manuscript:

* A rebuttal letter that responds to each point raised by the editor and reviewer(s). You should upload this letter as a separate file labeled 'Response to Reviewers'. This file does not need to include responses to any formatting updates and technical items listed in the 'Journal Requirements' section below.

* A marked-up copy of your manuscript that highlights changes made to the original version. You should upload this as a separate file labeled 'Revised Manuscript with Track Changes'.

* An unmarked version of your revised paper without tracked changes. You should upload this as a separate file labeled 'Manuscript'.

If you would like to make changes to your financial disclosure, competing interests statement, or data availability statement, please make these updates within the submission form at the time of resubmission. Guidelines for resubmitting your figure files are available below the reviewer comments at the end of this letter.

We look forward to receiving your revised manuscript.

Kind regards,

Haleh Ayatollahi

Section Editor

PLOS Digital Health

Haleh Ayatollahi

Section Editor

PLOS Digital Health

Leo Anthony Celi

Editor-in-Chief

PLOS Digital Health

orcid.org/0000-0001-6712-6626

**Journal Requirements:**

1. Please amend your detailed Financial Disclosure statement. This is published with the article. It must therefore be completed in full sentences and contain the exact wording you wish to be published.

i. State the initials, alongside each funding source, of each author to receive each grant.

ii. State what role the funders took in the study. If the funders had no role in your study, please state: “The funders had no role in study design, data collection and analysis, decision to publish, or preparation of the manuscript.”

2. Please provide separate figure files in .tif or .eps format.

For more information about figure files please see our guidelines:

https://journals.plos.org/digitalhealth/s/figures

https://journals.plos.org/digitalhealth/s/figures#loc-file-requirements

3. Please provide an Author Summary. This should appear in your manuscript between the Abstract (if applicable) and the Introduction, and should be 150–200 words long. The aim should be to make your findings accessible to a wide audience that includes both scientists and non-scientists. Sample summaries can be found on our website under Submission Guidelines:

https://journals.plos.org/digitalhealth/s/submission-guidelines#loc-parts-of-a-submission

4. We have noticed that you have uploaded Supporting Information files, but you have not included a list of legends. Please add a full list of legends for your Supporting Information files after the references list.

5. We note that your Data Availability Statement is currently as follows: All data are in the manuscript and supporting information files.

Please confirm at this time whether or not your submission contains all raw data required to replicate the results of your study. Authors must share the “minimal data set” for their submission. PLOS defines the minimal data set to consist of the data required to replicate all study findings reported in the article, as well as related metadata and methods (https://journals.plos.org/plosone/s/data-availability#loc-minimal-data-set-definition).

For example, authors should submit the following data:

- The values behind the means, standard deviations and other measures reported;

- The values used to build graphs;

- The points extracted from images for analysis.

Authors do not need to submit their entire data set if only a portion of the data was used in the reported study.

If your submission does not contain these data, please either upload them as Supporting Information files or deposit them to a stable, public repository and provide us with the relevant URLs, DOIs, or accession numbers. For a list of recommended repositories, please see https://journals.plos.org/plosone/s/recommended-repositories.

If there are ethical or legal restrictions on sharing a de-identified data set, please explain them in detail (e.g., data contain potentially sensitive information, data are owned by a third-party organization, etc.) and who has imposed them (e.g., an ethics committee). Please also provide contact information for a data access committee, ethics committee, or other institutional body to which data requests may be sent. If data are owned by a third party, please indicate how others may request data access.

If the reviewer comments include a recommendation to cite specific previously published works, please review and evaluate these publications to determine whether they are relevant and should be cited. There is no requirement to cite these works unless the editor has indicated otherwise.

**Additional Editor Comments (if provided):**

[Note: HTML markup is below. Please do not edit.]

**Reviewers' Comments:**

Reviewer's Responses to Questions

**Comments to the Author**

1. Does this manuscript meet PLOS Digital Health’s publication criteria? Is the manuscript technically sound, and do the data support the conclusions? The manuscript must describe methodologically and ethically rigorous research with conclusions that are appropriately drawn based on the data presented.

Reviewer #1: Yes

Reviewer #2: Yes

Reviewer #3: Partly

**********

2. Has the statistical analysis been performed appropriately and rigorously?

Reviewer #1: Yes

Reviewer #2: N/A

Reviewer #3: N/A

**********

3. Have the authors made all data underlying the findings in their manuscript fully available (please refer to the Data Availability Statement at the start of the manuscript PDF file)?

The PLOS Data policy requires authors to make all data underlying the findings described in their manuscript fully available without restriction, with rare exception. The data should be provided as part of the manuscript or its supporting information, or deposited to a public repository. For example, in addition to summary statistics, the data points behind means, medians and variance measures should be available. If there are restrictions on publicly sharing data—e.g. participant privacy or use of data from a third party—those must be specified.

Reviewer #1: Yes

Reviewer #2: Yes

Reviewer #3: No

**********

4. Is the manuscript presented in an intelligible fashion and written in standard English?

PLOS Digital Health does not copyedit accepted manuscripts, so the language in submitted articles must be clear, correct, and unambiguous. Any typographical or grammatical errors should be corrected at revision, so please note any specific errors here.

Reviewer #1: Yes

Reviewer #2: Yes

Reviewer #3: Yes

**********

5. Review Comments to the Author

Please use the space provided to explain your answers to the questions above. You may also include additional comments for the author, including concerns about dual publication, research ethics, or publication ethics. (Please upload your review as an attachment if it exceeds 20,000 characters)

Reviewer #1: General Comments

The manuscript entitled “A Scoping Review of Barriers and Facilitators to Implementing Tele-Mental Health Solutions for Rural, Remote and Underserved Populations in Low- and Middle-Income Countries” addresses a highly relevant and timely topic in global mental health. It targets an evident research gap concerning the deployment of digital mental health interventions in low-resource settings. The manuscript is generally well-structured and clearly written. The methodological approach, especially the use of the COM-B model, is appropriate and provides a valuable framework for categorising barriers and facilitators.

However, certain aspects of the study could be further strengthened. For example, providing more detail regarding the limitations of the search strategy, elaborating on the contextual nuances of the LMIC definition, and offering deeper discussion on gender dynamics or broader social inequities would enhance the manuscript’s contribution. While the language is mostly clear and academic, several sections could benefit from careful language polishing to improve precision and readability. Overall, I consider this a promising and relevant manuscript that can make a significant contribution to the field of digital health, provided the authors address the points raised in this review.

Major Comments

1. The manuscript focuses on LMICs; however, the term “low- and middle-income countries” encompasses highly heterogeneous contexts in terms of economy, culture, and health systems. I suggest that the authors discuss in greater depth which regions are represented in the included studies and to what extent the findings can be generalized to LMICs as a whole.

2. While the search strategy is clearly presented, it raises the question of whether sufficient databases were included, particularly those specific to LMIC contexts (e.g., LILACS, AJOL, or regional databases). I recommend that the authors justify their choice of databases and discuss what types of studies might have been missed due to this limitation.

3. The manuscript briefly mentions sociodemographic factors, but the discussion of gender-related issues and broader social inequalities remains superficial. I encourage the authors to elaborate on how these factors impact access to tele-mental health services in LMIC settings.

4. The Limitations section is relatively brief. I recommend that the authors expand on potential biases, such as publication bias, language bias (given that only English-language studies were included), and any limitations of the COM-B model when applied across diverse cultural contexts.

5. While the use of the COM-B model is an excellent choice, it remains unclear how exactly the categorisation was conducted. For example, how many researchers performed the coding, and how was interrater reliability ensured? I suggest that the authors provide more details about the coding process.

6. The study references various tele-mental health interventions (e.g., telephone counseling, video consultations, mobile apps), but it remains unclear which barriers or facilitators are more strongly associated with each type of intervention. I suggest that the authors provide a more nuanced discussion distinguishing which challenges pertain to specific service modalities.

7. While the manuscript’s conclusion is valuable, it primarily contains general statements. I encourage the authors to develop more concrete practical recommendations (e.g., implementation strategies, policy suggestions) for decision-makers and practitioners, particularly relevant for LMIC contexts.

Minor comments

1. Abstract. Replace “poses a great global public health concern” with the more academic phrasing: “represents a significant global public health concern.”

2. Abstract. Consider simplifying and splitting this long sentence: “Tele-mental health, which involves remote access to care through information and communications technologies (ICTs) and mobile communication systems, may represent a promising solution.”

3. Introduction. Insert hyphen in compound number: “Eighty-two percent of the global population struggling with at least one diagnosable health condition live in LMICs.”

4. Introduction. In standard academic writing, “social determinants of health” is usually lowercase unless referring to a specific framework. Change to: “social determinants of health such as low income, low education, and social exclusion are key contributors to mental health challenges.”

5. Introduction. “Access to appropriate mental health care remains a challenge in LMICs for various reasons.” Here, consider the more precise academic phrase: “continues to be a significant challenge in LMICs for various reasons.”

6. Methods. “Publications in English, Dutch, Cantonese Chinese, Spanish, and French were considered as our review team comprised individuals from diverse backgrounds who collectively spoke these languages.” Split this long sentence for clarity: “Publications in English, Dutch, Cantonese Chinese, Spanish, and French were considered. Our review team comprised individuals from diverse backgrounds who collectively spoke these languages.”

7. Discussion. “emerges as a promising alternative” could be more formal: “is positioned as a potentially effective alternative.”

Reviewer #2: Thank you for the opportunity to review this well-written article. This is a valuable piece of work which reviews the barriers and facilitators (for both service users and service providers) to online mental health support in low- and middle-income countries (LMICs). The barriers and facilitators identified are specific to their contexts in LMICs, as are the recommendations for future practice and policy. This detailed review will be useful for policymakers and future researchers in this field, so I recommend acceptance of this article, with some very minor revisions suggested based on the feedback below.

Feedback

The introduction should include a definition of LMICs, especially for an audience interested in Digital Health who may not be familiar with this terminology. The World Bank definition is mentioned in the inclusion criteria, but not explained. This could be defined at the outset.

Publications were considered in English, Dutch, Cantonese Chinese, Spanish, and French. Even though all included publications were in English, it is a strength of the study that the authors were able to consider a wide range of publications.

The inclusion criteria lists “Remote, rural and/or underserved communities”, but it is not clear how “underserved communities” were defined. Further clarity here would be appreciated.

The text states that 147 studies were excluded after abstract screening and 47 excluded after full-text screening. The reasons for exclusion are not stated in-text, but they are stated in Figure 1. Signposting this to the reader would be helpful.

The results section is clear and well-structured. The discussion section summarises the findings well and links to broader issues, such as the prevalence of other mental health issues like bipolar disorder, but a lack of TMHIs for these.

The implications for practice and policy section introduces concepts which need to be explained better for the reader. Healthcare 4.0 and Society 5.0 are referred to but should be explained and referenced. The term “chrono-policy levels” is introduced but it is not clear what this means. Finally, “NGO” should be spelled out instead of using an acronym.

Reviewer #3: I enjoyed reading your draft because of how nicely clearly written and how timely your research quetsion is, thanks very much for taking the time to do this research, it is meaningful.

Here is my take on the publication criteria: my comments on your draft is attached as pdf.

Originality - High importance and broad interest to community of researchers, engineers and clinicians working in the field of digital health

Methods: moderate methodological rigor and ethical standards. I think you should have reviwed the abstracts by skimming to confirm that exculded studies were accurately excluded. It is surprising that only 9 studies were found although the geographical areas are broad. I think once you address this part of your review, then you will jump to high methodlogical rigor. This is the main reason I chose "Major revision" in my assessment because study titles not always tell about research questions.

Substantial evidence for its conclusions - I think this is moderate too, maybe you can discuss more literature on the digitalhealth disparties in these regions or the lack of access to traditional health services so that you lay the ground for more robust conclusions beyond the 9 studies. and also if you include a "furture directions" paragraph that has some evience-based recommendations published in other studies in other regions that would further strengthen your paper.

Clearly outlined utility and accessibility for the broader community - I think this was done ok overall, but if you integrate the above feedback on methods and substantial evidence for its conclusions section, then you will get better in this section as well.

Follow appropriate standards and practice of open science - I think you did a good job and writing an easily replicable study, but there was no link to the original database list of studies for people to take a look. Yo have included a search prompt only, which is good, but not enough to have this fully open science.

**********

6. PLOS authors have the option to publish the peer review history of their article (what does this mean?). If published, this will include your full peer review and any attached files.

**Do you want your identity to be public for this peer review?** If you choose “no”, your identity will remain anonymous but your review may still be made public.

For information about this choice, including consent withdrawal, please see our Privacy Policy.

Reviewer #1: No

Reviewer #2: No

Reviewer #3: No

**********

[NOTE: If reviewer comments were submitted as an attachment file, they will be attached to this email and accessible via the submission site. Please log into your account, locate the manuscript record, and check for the action link "View Attachments". If this link does not appear, there are no attachment files.]

**Figure resubmission:**

While revising your submission, please upload your figure files to the Preflight Analysis and Conversion Engine (PACE) digital diagnostic tool, https://pacev2.apexcovantage.com/. PACE helps ensure that figures meet PLOS requirements. To use PACE, you must first register as a user. Registration is free. Then, login and navigate to the UPLOAD tab, where you will find detailed instructions on how to use the tool. If you encounter any issues or have any questions when using PACE, please email PLOS at figures@plos.org. Please note that Supporting Information files do not need this step. If there are other versions of figure files still present in your submission file inventory at resubmission, please replace them with the PACE-processed versions.

**Reproducibility:**

To enhance the reproducibility of your results, we recommend that authors of applicable studies deposit laboratory protocols in protocols.io, where a protocol can be assigned its own identifier (DOI) such that it can be cited independently in the future. Additionally, PLOS ONE offers an option to publish peer-reviewed clinical study protocols. Read more information on sharing protocols at https://plos.org/protocols?utm_medium=editorial-email&utm_source=authorletters&utm_campaign=protocols

Attachment Submitted filename: PDIG-D-25-00312_reviewer comments final.pdf

# Author comment: Author response to Decision Letter 1

**DOI:** 10.1371/journal.pdig.0000903.r002

21 Oct 2025

Attachment Submitted filename: PLOS Digital Health responses to reviewer comments 14.10.25.docx

# Peer review: Decision Letter 1

**DOI:** 10.1371/journal.pdig.0000903.r003

**Authors:** Haleh Ayatollahi

7 Nov 2025

PDIG-D-25-00312R1

A scoping review of barriers and facilitators to implementing tele-mental health solutions for rural, remote and underserved populations in low- and middle-income countries

PLOS Digital Health

Dear Dr. Jumbe,

Thank you for submitting your manuscript to PLOS Digital Health. After careful consideration, we feel that it has merit but does not fully meet PLOS Digital Health's publication criteria as it currently stands. Therefore, we invite you to submit a revised version of the manuscript that addresses the points raised during the review process.

Please submit your revised manuscript by Dec 07 2025 11:59PM. If you will need more time than this to complete your revisions, please reply to this message or contact the journal office at digitalhealth@plos.org. When you're ready to submit your revision, log on to https://www.editorialmanager.com/pdig/ and select the 'Submissions Needing Revision' folder to locate your manuscript file.

Please include the following items when submitting your revised manuscript:

* A rebuttal letter that responds to each point raised by the editor and reviewer(s). You should upload this letter as a separate file labeled 'Response to Reviewers'. This file does not need to include responses to any formatting updates and technical items listed in the 'Journal Requirements' section below.

* A marked-up copy of your manuscript that highlights changes made to the original version. You should upload this as a separate file labeled 'Revised Manuscript with Track Changes'.

* An unmarked version of your revised paper without tracked changes. You should upload this as a separate file labeled 'Manuscript'.

If you would like to make changes to your financial disclosure, competing interests statement, or data availability statement, please make these updates within the submission form at the time of resubmission. Guidelines for resubmitting your figure files are available below the reviewer comments at the end of this letter.

We look forward to receiving your revised manuscript.

Kind regards,

Haleh Ayatollahi

Section Editor

PLOS Digital Health

Haleh Ayatollahi

Section Editor

PLOS Digital Health

Leo Anthony Celi

Editor-in-Chief

PLOS Digital Health

orcid.org/0000-0001-6712-6626

**Journal Requirements:**

If the reviewer comments include a recommendation to cite specific previously published works, please review and evaluate these publications to determine whether they are relevant and should be cited. There is no requirement to cite these works unless the editor has indicated otherwise.

**Additional Editor Comments (if provided):**

[Note: HTML markup is below. Please do not edit.]

**Reviewers' Comments:**

Reviewer's Responses to Questions

**Comments to the Author**

1. If the authors have adequately addressed your comments raised in a previous round of review and you feel that this manuscript is now acceptable for publication, you may indicate that here to bypass the “Comments to the Author” section, enter your conflict of interest statement in the “Confidential to Editor” section, and submit your "Accept" recommendation.

Reviewer #1: All comments have been addressed

Reviewer #2: All comments have been addressed

Reviewer #3: (No Response)

**********

2. Does this manuscript meet PLOS Digital Health’s publication criteria? Is the manuscript technically sound, and do the data support the conclusions? The manuscript must describe methodologically and ethically rigorous research with conclusions that are appropriately drawn based on the data presented.

Reviewer #1: Yes

Reviewer #2: Yes

Reviewer #3: Partly

**********

3. Has the statistical analysis been performed appropriately and rigorously?

Reviewer #1: Yes

Reviewer #2: N/A

Reviewer #3: N/A

**********

4. Have the authors made all data underlying the findings in their manuscript fully available (please refer to the Data Availability Statement at the start of the manuscript PDF file)?

The PLOS Data policy requires authors to make all data underlying the findings described in their manuscript fully available without restriction, with rare exception. The data should be provided as part of the manuscript or its supporting information, or deposited to a public repository. For example, in addition to summary statistics, the data points behind means, medians and variance measures should be available. If there are restrictions on publicly sharing data—e.g. participant privacy or use of data from a third party—those must be specified.

Reviewer #1: Yes

Reviewer #2: Yes

Reviewer #3: Yes

**********

5. Is the manuscript presented in an intelligible fashion and written in standard English?

PLOS Digital Health does not copyedit accepted manuscripts, so the language in submitted articles must be clear, correct, and unambiguous. Any typographical or grammatical errors should be corrected at revision, so please note any specific errors here.

Reviewer #1: Yes

Reviewer #2: Yes

Reviewer #3: No

**********

6. Review Comments to the Author

Please use the space provided to explain your answers to the questions above. You may also include additional comments for the author, including concerns about dual publication, research ethics, or publication ethics. (Please upload your review as an attachment if it exceeds 20,000 characters)

Reviewer #1: General comments

The revised manuscript demonstrates substantial improvement and reflects the authors’ thorough engagement with the reviewers’ feedback. The inclusion of detailed methodological clarifications (e.g., coding process, COM-B model application) and the expanded discussion on social and gender inequities have significantly enhanced both transparency and depth. Conceptual clarifications, such as the definition of LMICs and “underserved” communities, further strengthen the paper’s precision and readability. Overall, the manuscript represents a valuable contribution to understanding global inequities in tele-mental health implementation and offers practical insights for policy and practice. I have only two additional major recommendations before final acceptance, both intended to enhance coherence and policy relevance.

Specific comments

1. While the revised manuscript offers a clear regional overview, it would benefit from a brief integrative synthesis that identifies which barriers and facilitators appear universal across LMICs and which are region-specific. Adding a concise paragraph to the end of the Discussion or Implications for Policy section could strengthen cross-context comparability and enhance the paper’s relevance for global health decision-makers.

2. The conclusions outline meaningful future directions but remain somewhat general. I recommend specifying which types of research approaches (e.g., realist reviews, participatory implementation studies, mixed-methods longitudinal designs) or partnership models (e.g., NGO–academic collaborations) could most effectively advance sustainable tele-mental health implementation in LMICs. Such specification would enhance the practical value of the manuscript and provide clearer guidance for future research.

Reviewer #2: Thank you for the opportunity to review the revised manuscript. All my previous comments have been addressed.

Reviewer #3: Thank you very much for addressing many of my comments and feedback. You missed many of my comments on the attached pdf I have provided and here they are again as a response to your response.

Line 148 Table 2: Search strategies used within the scoping review : you did not address my comment on the definition of mental health intervention. A mental health intervention could be for a physical condition like behavioral medicine services, was that included in the search or excluded? That should be clarified.

Line 150 Screening and selection of evidence - I suggested spilling out the names of reviewers so that the reader knows what the letters stand for, is this someone external or representing the names of authors basically.

Line 306 and 307 not sure you fixed the redundancy in phrases I noted in my comments?

Line 443 the phrase “For providers, common key facilitators related to reflective motivation.” is very confusing thrown in there without clear connection to context or paragraph, I suggest rewriting it.

Line 459 the limitations section: you did not address my comment on the pdf about defining the full spectrum of what is considered mental health interventions and whether you searched for lifestyle psychological interventions (i.e. behavioral medicine interventions) or not? This distinction is very important and it has to be described in the limitations section if you did not include it in the search which could lead to excluding unintentionally studies that could be fitting the criteria.

Line 475 I also have a comment on the pdf about the word “At chrono-policy levels” is not clear for me as a scholar and how about the general reader. Please define these terms in a glossary if not inside the text of the paragraph. Accessibility of terminology and jargon free papers are important for open science and accessibility of knowledge.

Line 479 I also commented on the pdf about adding some info about the economic burden of mental health problems and the lack of access in these communities so that your statement is more persuasive and effective.

Line 511 I commented on the pdf about adding the WHO guidelines or policies on digital health which is also very relevant here and it is more persuasive to add a global health agency’s publication rather than a state-specific only like the US’s or the UK’s or Canada’s policies.

Line 468 Implications for Practice and Policy : you did not add any of the future questions that should be answered in future studies in order to overcome the limitations in yours (AKA the things you have missed in this study and you hopefully described in the limitations section, now you have to mention the need for studying or addressing these limitations in future studies.

Finally, you have indicated in your response to my comment 3 as you see below in the paragraph that there were additions to the conclusion or discussion or future implications sections, but I couldn’t find anything about integrating the information I asked for in my comment in any of your sections nor the lines you have indicated except for citations. Are you missing something here? Or you have added only citations? It should be discussed in the sections described clearly, not only in citations.

Here is your response that I am referring to: “Response to reviewer 3 comment 3: Noted. We have revised the conclusion (lines 600-619) to include “future directions" within paragraph as based on evidence-based recommendations from published studies we have added to discussion following reviewers’ comments as well as findings from our WUN collaborative project case studies that will be published in the near future.”

**********

7. PLOS authors have the option to publish the peer review history of their article (what does this mean?). If published, this will include your full peer review and any attached files.

**Do you want your identity to be public for this peer review?** If you choose “no”, your identity will remain anonymous but your review may still be made public.

For information about this choice, including consent withdrawal, please see our Privacy Policy.

Reviewer #1: No

Reviewer #2: No

Reviewer #3: No

**********

[NOTE: If reviewer comments were submitted as an attachment file, they will be attached to this email and accessible via the submission site. Please log into your account, locate the manuscript record, and check for the action link "View Attachments". If this link does not appear, there are no attachment files.]

**Figure resubmission:**

While revising your submission, we strongly recommend that you use PLOS’s NAAS tool (https://ngplosjournals.pagemajik.ai/artanalysis) to test your figure files. NAAS can convert your figure files to the TIFF file type and meet basic requirements (such as print size, resolution), or provide you with a report on issues that do not meet our requirements and that NAAS cannot fix.

After uploading your figures to PLOS’s NAAS tool - https://ngplosjournals.pagemajik.ai/artanalysis, NAAS will process the files provided and display the results in the "Uploaded Files" section of the page as the processing is complete. If the uploaded figures meet our requirements (or NAAS is able to fix the files to meet our requirements), the figure will be marked as "fixed" above. If NAAS is unable to fix the files, a red "failed" label will appear above. When NAAS has confirmed that the figure files meet our requirements, please download the file via the download option, and include these NAAS processed figure files when submitting your revised manuscript.

**Reproducibility:**

To enhance the reproducibility of your results, we recommend that authors of applicable studies deposit laboratory protocols in protocols.io, where a protocol can be assigned its own identifier (DOI) such that it can be cited independently in the future. Additionally, PLOS ONE offers an option to publish peer-reviewed clinical study protocols. Read more information on sharing protocols at https://plos.org/protocols?utm_medium=editorial-email&utm_source=authorletters&utm_campaign=protocols

# Author comment: Author response to Decision Letter 2

**DOI:** 10.1371/journal.pdig.0000903.r004

17 Dec 2025

Attachment Submitted filename: PLOS Digital Health responses to reviewer comments 10.12.25.docx

# Peer review: Decision Letter 2

**DOI:** 10.1371/journal.pdig.0000903.r005

**Authors:** Haleh Ayatollahi

26 Feb 2026

PDIG-D-25-00312R2

A scoping review of barriers and facilitators to implementing tele-mental health solutions for rural, remote and underserved populations in low- and middle-income countries

PLOS Digital Health

Dear Dr. Jumbe,

Thank you for submitting your manuscript to PLOS Digital Health. After careful consideration, we feel that it has merit but does not fully meet PLOS Digital Health's publication criteria as it currently stands. Therefore, we invite you to submit a revised version of the manuscript that addresses the points raised during the review process.

Please submit your revised manuscript by Mar 28 2026 11:59PM. If you will need more time than this to complete your revisions, please reply to this message or contact the journal office at digitalhealth@plos.org. When you're ready to submit your revision, log on to https://www.editorialmanager.com/pdig/ and select the 'Submissions Needing Revision' folder to locate your manuscript file.

Please include the following items when submitting your revised manuscript:

* A letter that responds to each point raised by the editor and reviewer(s). You should upload this letter as a separate file labeled 'Response to Reviewers'. This file does not need to include responses to any formatting updates and technical items listed in the 'Journal Requirements' section below.

* A marked-up copy of your manuscript that highlights changes made to the original version. You should upload this as a separate file labeled 'Revised Manuscript with Track Changes'.

* An unmarked version of your revised paper without tracked changes. You should upload this as a separate file labeled 'Manuscript'.

If you would like to make changes to your financial disclosure, competing interests statement, or data availability statement, please make these updates within the submission form at the time of resubmission. Guidelines for resubmitting your figure files are available below the reviewer comments at the end of this letter.

We look forward to receiving your revised manuscript.

Kind regards,

Haleh Ayatollahi

Section Editor

PLOS Digital Health

Haleh Ayatollahi

Section Editor

PLOS Digital Health

Leo Anthony Celi

Editor-in-Chief

PLOS Digital Health

orcid.org/0000-0001-6712-6626

**Journal Requirements:**

If the reviewer comments include a recommendation to cite specific previously published works, please review and evaluate these publications to determine whether they are relevant and should be cited. There is no requirement to cite these works unless the editor has indicated otherwise.

**Additional Editor Comments (if provided):**

[Note: HTML markup is below. Please do not edit.]

**Reviewers' Comments:**

Reviewer's Responses to Questions

**Comments to the Author**

1. If the authors have adequately addressed your comments raised in a previous round of review and you feel that this manuscript is now acceptable for publication, you may indicate that here to bypass the “Comments to the Author” section, enter your conflict of interest statement in the “Confidential to Editor” section, and submit your "Accept" recommendation.

Reviewer #1: All comments have been addressed

Reviewer #3: (No Response)

**********

2. Does this manuscript meet PLOS Digital Health’s publication criteria? Is the manuscript technically sound, and do the data support the conclusions? The manuscript must describe methodologically and ethically rigorous research with conclusions that are appropriately drawn based on the data presented.

Reviewer #1: Yes

Reviewer #3: Yes

**********

3. Has the statistical analysis been performed appropriately and rigorously?

Reviewer #1: Yes

Reviewer #3: N/A

**********

4. Have the authors made all data underlying the findings in their manuscript fully available (please refer to the Data Availability Statement at the start of the manuscript PDF file)?

The PLOS Data policy requires authors to make all data underlying the findings described in their manuscript fully available without restriction, with rare exception. The data should be provided as part of the manuscript or its supporting information, or deposited to a public repository. For example, in addition to summary statistics, the data points behind means, medians and variance measures should be available. If there are restrictions on publicly sharing data—e.g. participant privacy or use of data from a third party—those must be specified.

Reviewer #1: Yes

Reviewer #3: (No Response)

**********

5. Is the manuscript presented in an intelligible fashion and written in standard English?

PLOS Digital Health does not copyedit accepted manuscripts, so the language in submitted articles must be clear, correct, and unambiguous. Any typographical or grammatical errors should be corrected at revision, so please note any specific errors here.

Reviewer #1: Yes

Reviewer #3: Yes

**********

6. Review Comments to the Author

Please use the space provided to explain your answers to the questions above. You may also include additional comments for the author, including concerns about dual publication, research ethics, or publication ethics. (Please upload your review as an attachment if it exceeds 20,000 characters)

Reviewer #1: Thank you for the opportunity to review the revised manuscript.

All my previous comments have been addressed

Reviewer #3: thanks for addressing my comments, there is one more. You will see it in the attached pdf on page 8, response to comment 5 of reviewer 3. I still think it would be better to mention the distinction between mental health intervention versus behavioral medicine intervention in the eligibility criteria section because the paper should be clear and transparent on its eligibility criteria and you don't want to mislead readers that those are included. transparency is good for you and for facilitating future directions based on the limitations of this study. In your response, you explained to me but did not explain that to the reader in the manuscript.

**********

7. PLOS authors have the option to publish the peer review history of their article (what does this mean?). If published, this will include your full peer review and any attached files.

**Do you want your identity to be public for this peer review?** If you choose “no”, your identity will remain anonymous but your review may still be made public.

For information about this choice, including consent withdrawal, please see our Privacy Policy.

Reviewer #1: No

Reviewer #3: No

**********

[NOTE: If reviewer comments were submitted as an attachment file, they will be attached to this email and accessible via the submission site. Please log into your account, locate the manuscript record, and check for the action link "View Attachments". If this link does not appear, there are no attachment files.]

**Figure resubmission:**

While revising your submission, we strongly recommend that you use PLOS’s NAAS tool (https://ngplosjournals.pagemajik.ai/artanalysis) to test your figure files. NAAS can convert your figure files to the TIFF file type and meet basic requirements (such as print size, resolution), or provide you with a report on issues that do not meet our requirements and that NAAS cannot fix.

After uploading your figures to PLOS’s NAAS tool - https://ngplosjournals.pagemajik.ai/artanalysis, NAAS will process the files provided and display the results in the "Uploaded Files" section of the page as the processing is complete. If the uploaded figures meet our requirements (or NAAS is able to fix the files to meet our requirements), the figure will be marked as "fixed" above. If NAAS is unable to fix the files, a red "failed" label will appear above. When NAAS has confirmed that the figure files meet our requirements, please download the file via the download option, and include these NAAS processed figure files when submitting your revised manuscript.

**Reproducibility:**

To enhance the reproducibility of your results, we recommend that authors of applicable studies deposit laboratory protocols in protocols.io, where a protocol can be assigned its own identifier (DOI) such that it can be cited independently in the future. Additionally, PLOS ONE offers an option to publish peer-reviewed clinical study protocols. Read more information on sharing protocols at https://plos.org/protocols?utm_medium=editorial-email&utm_source=authorletters&utm_campaign=protocols

Attachment Submitted filename: PDIG-D-25-00312_R2_reviewer (1).pdf

# Author comment: Author response to Decision Letter 3

**DOI:** 10.1371/journal.pdig.0000903.r006

6 Mar 2026

Attachment Submitted filename: PLOS Digital Health responses to reviewer comments 6.3.26.docx

# Peer review: Decision Letter 3

**DOI:** 10.1371/journal.pdig.0000903.r007

**Authors:** Haleh Ayatollahi

8 May 2026

A scoping review of barriers and facilitators to implementing tele-mental health solutions for rural, remote and underserved populations in low- and middle-income countries

PDIG-D-25-00312R3

Dear Dr Jumbe,

We are pleased to inform you that your manuscript 'A scoping review of barriers and facilitators to implementing tele-mental health solutions for rural, remote and underserved populations in low- and middle-income countries' has been provisionally accepted for publication in PLOS Digital Health.

Before your manuscript can be formally accepted you will need to complete some formatting changes, which you will receive in a follow-up email from a member of our team.

Please note that your manuscript will not be scheduled for publication until you have made the required changes, so a swift response is appreciated.

IMPORTANT: The editorial review process is now complete. PLOS will only permit corrections to spelling, formatting or significant scientific errors from this point onwards. Requests for major changes, or any which affect the scientific understanding of your work, will cause delays to the publication date of your manuscript.

If your institution or institutions have a press office, please notify them about your upcoming paper to help maximize its impact. If they'll be preparing press materials, please inform our press team as soon as possible -- no later than 48 hours after receiving the formal acceptance. Your manuscript will remain under strict press embargo until 2 pm Eastern Time on the date of publication. For more information, please contact digitalhealth@plos.org.

Thank you again for supporting Open Access publishing; we are looking forward to publishing your work in PLOS Digital Health.

Best regards,

Haleh Ayatollahi

Section Editor

PLOS Digital Health

***********************************************************

**Additional Editor Comments (if provided):**

**Reviewer Comments (if any, and for reference):**

Reviewer's Responses to Questions

**Comments to the Author**

1. If the authors have adequately addressed your comments raised in a previous round of review and you feel that this manuscript is now acceptable for publication, you may indicate that here to bypass the “Comments to the Author” section, enter your conflict of interest statement in the “Confidential to Editor” section, and submit your "Accept" recommendation.

Reviewer #3: All comments have been addressed

**********

2. Does this manuscript meet PLOS Digital Health’s publication criteria? Is the manuscript technically sound, and do the data support the conclusions? The manuscript must describe methodologically and ethically rigorous research with conclusions that are appropriately drawn based on the data presented.

Reviewer #3: Yes

**********

3. Has the statistical analysis been performed appropriately and rigorously?

Reviewer #3: (No Response)

**********

4. Have the authors made all data underlying the findings in their manuscript fully available (please refer to the Data Availability Statement at the start of the manuscript PDF file)?

The PLOS Data policy requires authors to make all data underlying the findings described in their manuscript fully available without restriction, with rare exception. The data should be provided as part of the manuscript or its supporting information, or deposited to a public repository. For example, in addition to summary statistics, the data points behind means, medians and variance measures should be available. If there are restrictions on publicly sharing data—e.g. participant privacy or use of data from a third party—those must be specified.

Reviewer #3: (No Response)

**********

5. Is the manuscript presented in an intelligible fashion and written in standard English?

PLOS Digital Health does not copyedit accepted manuscripts, so the language in submitted articles must be clear, correct, and unambiguous. Any typographical or grammatical errors should be corrected at revision, so please note any specific errors here.

Reviewer #3: Yes

**********

6. Review Comments to the Author

Please use the space provided to explain your answers to the questions above. You may also include additional comments for the author, including concerns about dual publication, research ethics, or publication ethics. (Please upload your review as an attachment if it exceeds 20,000 characters)

Reviewer #3: Thank you very much for your professionalism and valuing our comments and again on working on this important topic.

**********

7. PLOS authors have the option to publish the peer review history of their article (what does this mean?). If published, this will include your full peer review and any attached files.

**Do you want your identity to be public for this peer review?** If you choose “no”, your identity will remain anonymous but your review may still be made public.

For information about this choice, including consent withdrawal, please see our Privacy Policy.

Reviewer #3: No

**********
